# Changes on proteomic and metabolomic profile in serum of mice induced by chronic exposure to tramadol

**DOI:** 10.1038/s41598-021-81109-7

**Published:** 2021-01-14

**Authors:** Shukun Jiang, Guojie Liu, Huiya Yuan, Enyu Xu, Wei Xia, Xiaoyu Zhang, Junting Liu, Lina Gao

**Affiliations:** 1 School of Forensic Medicine, China Medical University, No. 77, Puhe Road, Shenyang North New Area, Shenyang, 110122 Liaoning Province People’s Republic of China; 2 School of Fundamental Sciences, China Medical University, Shenyang, 110014 People’s Republic of China; 3 Jacobs University, 28759 Bremen, Germany

**Keywords:** Metabolomics, Proteomics

## Abstract

Tramadol is an opioid used as an analgesic for treating moderate or severe pain. The long-term use of tramadol can induce several adverse effects. The toxicological mechanism of tramadol abuse is unclear. Limited literature available indicates the change of proteomic profile after chronic exposure to tramadol. In this study, we analyzed the proteomic and metabolomic profile by TMT-labeled quantitative proteomics and untargeted metabolomics between the tramadol and the control group. Proteomic analysis revealed 31 differential expressed serum proteins (9 increased and 22 decreased) in tramadol-treated mice (oral, 50 mg/kg, 5 weeks) as compared with the control ones. Bioinformatics analysis showed that the dysregulated proteins mainly included: enzyme inhibitor-associated proteins (i.e. apolipoprotein C-III (Apoc-III), alpha-1-antitrypsin 1–2 (Serpina 1b), apolipoprotein C-II (Apoc-II), plasma protease C1 inhibitor, inter-alpha-trypsin inhibitor heavy chain H3 (itih3)); mitochondria-related proteins (i.e. 14-3-3 protein zeta/delta (YWHAZ)); cytoskeleton proteins (i.e. tubulin alpha-4A chain (TUBA4A), vinculin (Vcl)). And we found that the differential expressed proteins mainly involved in the pathway of the protein digestion and absorption. Metabolomics analysis revealed that differential expressed metabolites mainly involved in protein ingestion and absorption, fatty acid biosynthesis, steroid hormone biosynthesis and bile secretion. Our overall findings revealed that chronic exposure to tramadol changed the proteomic and metabolomic profile of mice. Moreover, integrated proteomic and metabolomic revealed that the protein digestion and absorption is the common enrichment KEGG pathway. Thus, the combination of proteomics and metabolomics opens new avenues for the research of the molecular mechanisms of tramadol toxicity.

## Introduction

Tramadol is a central analgesic drug and a low-affinity opioid receptor agonist, which is often used for the treatment of moderate to severe pain^1,2^. Tramadol is metabolized into O-demethyltramadol and N-demethyltramadol, of which O-demethyltramadol has stronger pharmacological activity than the original drug^3^. Tramadol has analgesic and other functions by acting in two ways: O-demethylated metabolites with bio activity, and the synergistic effects of opioid and non-opioid mechanisms^4^.

Although tramadol has been an effective and well-tolerated agent for the management of moderately several acute or chronic pain^3^, its adverse effects have always been a concern for researchers. The untoward effects of tramadol include confusion, dizziness, seizures, drowsiness, and respiratory depression^4,5^. Especially after a long period of high doses of tramadol, more severe side effects include angioedema, the increased effect of anticoagulants, hypoglycemia^6,7^ and serotonin toxicity^1^. Some literature revealed that opioids abuse may result in structural changes and apoptosis of neurons^8,9^. Zhou had pointed out that chronic exposure to tramadol could induce toxic effect to the neurotransmitters of zebrafish^10^. Mohamed reported that chronic exposure to tramadol induced oxidative damage, inflammation, and apoptosis on the cerebrum of rats^4^. To the best of our knowledge, some literature have revealed that chronic tramadol administration is related to the provoking production of reactive oxygen species (ROS)^11–13^.

Metabolomics and proteomics, as components of systems biology, help to analyze the tramadol toxic mechanism from the perspective of organism integrity^14,15^. Moreover, the advent of advanced quantitative proteomics techniques allows the systematic study of changes in the expression profile of proteins that are static or perturbed^15,16^.

Although brain tissues are the best samples for laboratory research on central nervous system disorders such as addiction, blood can be collected much more easily and at much lower risks^17^. Importantly, it contains nearly the entire proteome of the human body^17^. Moreover, the effects of long term exposure to tramadol on protein and metabolite in serum has not been known.To mimic the long exposure to tramadol, mice were exposed to 50 mg/kg level of tramadol for 35 days. Blood plasma was then used for tandem mass tags (TMT) quantitative proteomics and untargeted metabolomics. In the present study, we aim to observe the proteomic and metabolomic profile to provide the fundamental for the tramadol toxicity in serum.

## Materials and methods

### Chemical reagents

Tramadol was purchased from Shanghai Macklin Biotechnology Co., Ltd. (Shanghai, China). LC–MS/MS testing was conducted by Shanghai Bioprofile Technology Co. Ltd. Mouse Tubulin Alpha 4A (TUBa4A) ELISA kit, mouse Vinculin (Vcl) ELISA kit, mouse Inter-alpha-trypsin inhibitor heavy chain H3 (itih3) ELISA kit, Mouse Alpha-1-antitrypsin 1–2 (Serpina1b) ELISA kit, Mouse Transthyretin (Ttr) ELISA kit, Mouse Haptoglobin (Hp) ELISA kit, Mouse Retinol-binding protein 4 (RBP-4) ELISA kit, Mouse Apolipoprotein C-III (ApocIII) ELISA kit, Mouse Plasma protease C1 inhibitor (Serping) ELISA kit, Mouse Carboxypeptidase B2 (CPB2) ELISA kit, Mouse 14-3-3 protein zeta/delta (YWHAZ) ELISA kit, and Mouse Hemopexin (Hpx) ELISA kits were purchased from Shanghai Enzyme-linked Biotechnology Co., Ltd. (Shanghai, China).

### Animal treatment and sample collection

16 km male mice (6 weeks old, 35 ± 5 g, Laboratory Animal Centre of China Medical University) were used in the studies. The mice were randomly assigned to a tramadol group (H group, 8 mice) or control group (N group, eight mice) and were housed in a controlled environment (20–22 °C; 12-h light:dark on a reversed light cycle) for 1 week before the studies. Mice had unlimited access to water and food in their home cages. Mice received physiological saline (20 mL/kg) or tramadol (2.5 mg/mL) via oral gavage daily (50 mg/kg/day) for 35 days in group. Mice were fasted for 24 h and anesthetized by intraperitoneal injection with pentobarbital prior to blood collection. Heart blood of each mouse was collected and centrifuged at 3000 rpm for 15 min at 4 °C to obtain the serum, for proteomics and metabolomics analysis.

### Serum proteomics analysis

#### Protein extraction

Approximately 100 μL of serum was taken from each sample group (the tramadol group and the control group), which was then added into 600 μL 8 M urea. Subsequently, sonicated, and the supernatant was extracted after centrifugation at 20,000 × *g* for 15 min at 4 ℃. Protein quantification was performed using the bicinchonic acid assay. About 15 μg of protein was obtained from the two different groups for SDS-PAGE analysis to evaluate the quantitative accuracy and quantify protein extraction.

#### Protein digestion and peptide desalination

300 μg of sample from each group was taken for protein digestion. DTT was added into the protein sample, and the final concentration was 100 mM. Bathe in boiling water for 5 min and cool to room temperature. After adding 200 μL of urea (UA) buffer (8 M UA and 150 mM Tris–HCl, pH 8.0), the samples were loaded on a 10 kDa ultra filtration centrifuge tube, followed by centrifugation at 12,000*g* for 15 min, and the filtrate was discarded (this step was repeated once). Subsequently, samples and 100 μL of iodoacetamide alkylation (IAA) (50 mm IAA in UA) were shaken for 1 min at 600 RPM, then were placed in a centrifuge at 12,000*g* × 10 min after 30 min at room temperature in the dark. Next, another 100 μL UA buffer was added, and the sample was centrifuged at 12,000*g* × 10 min. We repeated the process twice. This was followed by a 100 μL 100 mm NH_4_HCO_3_ buffer (Sigma), which was centrifuged at 14,000*g* for 10 min. We repeated the process twice. Next, 40 μL of trypsin buffer (6 µg trypsin in 40 µL NH_4_HCO_3_ buffer) were added into the sample. The sample was shaken at 600 RPM for 1 min and incubated at 37 °C for 16–18 h. The collection tube was replaced and then centrifuged for 10 min at 12,000*g*. The filtrate was collected and an amount of 0.1% trifluoroacetic acid (TFA) solution was added, followed by desalination in a C18 Cartridge (Sigma-Aldrich) and OD280 peptide quantification. Finally, approximately 150–180 µg peptides were collected.

#### TMT peptide labeling, fractionation and LC–MS/MS analysis

This part of the content was operated according to the method reported by Zhang^18^, and the specific method parameters are shown in “Supplement file”. The schematic for the TMT-labeling strategy used for the samples as shown in Supplementary Fig. 1.

#### Database search and protein quantification

Database search is similar with the method reported by Zhang^18^, the specific method parameters are shown in “Supplement file”. False discovery rate (FDR) for peptide and protein identification was set to 1%. The TMT reporter ion intensity was applied for quantification. The relative quantitative protein analysis of samples was performed using MaxQuant algorithms (http://www.maxquant.org, VERSION 1.6.0.16)^19^. The mass spectrometry proteomics data have been deposited to the ProteomeXchange Consortium (http://proteomecentral.proteomexchange.org) via the iProX partner repository with the dataset identifier PXD019233^20^.

#### Bioinformatics analysis

Analyses of bioinformatics data were obtained by the Perseus software program^21^, Microsoft Excel, and R statistical computing software. Differential expressed proteins (DEPs) were screened in the tramadol group and the control group, with the cut-off of ratio fold change of > 1.20 or < 0.83 in expression and P values < 0.05. In addition, DEPs in the tramadol group vs. the control groups were identified using student’s T-Test. The proteins with significant difference (FDR q < 0.01) were clustered by Hierarchical cluster analysis (Euclidean distance). Information was extracted from UniProtKB/Swiss-Prot, Kyoto Encyclopedia of Genes and genome (KEGG) and Gene Ontology (GO) for functional enrichment analysis of identified proteins^22,23^. Fisher's exact test was used for GO and KEGG enrichment analysis, and Benjamini–Hochberg false discovery rate (BH-FDR) correction for multiple tests was obtained. The term GO could be divided into three categories: biological process (BP), cellular component (CC), and molecular function (MF)^24^. The construction of PPI networks was also performed by the STRING database with the Cytoscape software program^25^.

### Untargeted metabolomics analysis

#### Metabolite extraction

100 μL of water and 800 μL of methanol/acetonitrile (1:1, v/v) were added for each sample (100 μL). Ultrasound was performed at low temperature for 30 min, twice, incubated at − 20 ℃ for 1 h, and centrifuged at 4 ℃ at 13,000 RPM for 15 min, the supernatant was taken for freeze-drying and stored at − 80 ℃ for later use. During mass spectrometry, 100 μL acetonitrile solution was added (acetonitrile: water = 1:1, v/v) was redissolved, vortex oscillated, centrifuged at 14,000*g* at 4 ℃ for 15 min, and the supernatant was injected into the LC–MS/MS system analysis.

#### Sample analysis and data preprocessing

This part was shown in “Supplement file”. The data preprocessing was performed according to the method reported by Gao^26^. The resulting matrix was imported into SIMCA-P (version 13.0, Umetrics, Sweden) for unsupervised principal component analysis (PCA) and orthogonal partial least square discriminant analysis (OPLS-DA)after mean centering and unit variance scaling. We applied uni-variate analysis (t-test) to calculate statistical significance (P-value). Metabolites with variable importance in the project (VIP) > 1, P-value < 0.05 were considered differential metabolites. Metabolites of interest were filtered based on values of VIP, |log2FC| > 1 (FC > 2 or FC < 0.5 and P-value < 0.05). Volcano plots were utilized to illustrate the distribution of differential metabolites.For clustering heat maps, data were normalized using z-scores of the intensity areas of differential metabolites and were plotted using the R package^27^ “pheatmap”.

#### Data quality evaluation in untargeted metabolomics analysis

Quality control samples (QCs) were obtained by pooling equal aliquots of each serum sample. QC samples are used to balance the chromatography-mass spectrometry system, to determine the state of the instrument, and to evaluate the stability of the system throughout the experiment. After mixing thoroughly, the QCs were analyzed consistently with real samples. Before the batch analysis, five QCs were first tested to stabilize the analytical system, and the acquired data were removed before data processing. All QCs were inserted randomly through the analytical batch to monitor the robustness of sample preparation and the stability of instrument analysis.

### Elisa

Carboxypeptidase B2 (CPB2), inter-alpha-trypsin inhibitor heavy chain H3 (Itih3), Alpha-1-antitrypsin1-2 (Serpina1b), plasma protease C1 inhibitor (serping), Tubulin alpha-4A chain (Tuba4a), Vinculin (Vcl) and apolipoprotein c-III (ApocIII), haptoglobin (Hp), Transthyretin (Ttr), Retinol-binding protein 4(RBP-4), 14-3-3 protein zeta/delta (YWHAZ), and Hemopexin (Hpx) Elisa kits were used to analyze the serum of ApocIII, Ttr, RBP-4,TUBa4A, Serpina1b, Vcl, Hp,YWHAZ, Hpx, CPB2, Itih3 and serping level following the manufacture’s instruction.

### Statistical analysis

The levels of ApocIII, Ttr, RBP-4, TUBa4A, Serpina1b, Vcl, Hp, YWHAZ, Hpx, CPB2, Itih3 and serping were present as mean ± standard deviation (SD) and analyzed by SPSS 26.0 (SPSS Inc., Chicago, USA). The student’s T-test was used and P < 0.05 was considered statistically significant.

### Ethics approval

All experimental procedures were conducted in accordance with the guidelines of the animal care institution and approved by the laboratory animal management committee of China Medical University (2019288).

## Results

### Proteomics results

**Figure 1:**
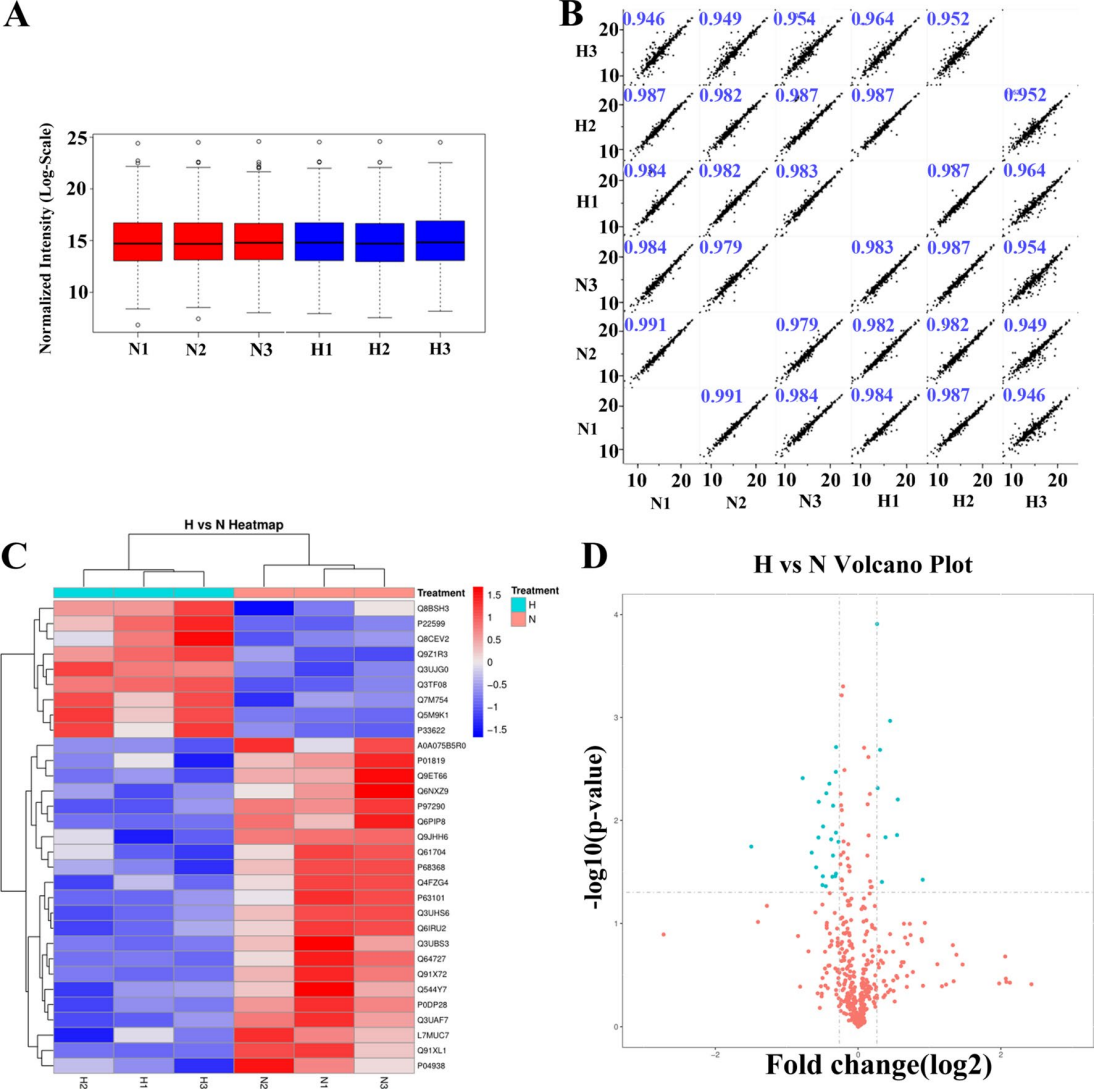
Volcano plots and heatmaps of proteins with differential expression between the tramadol group and the control group. (**A**) Boxplot of normalized density. (**B**) Pearson’s correlation of normalized densities; (**C**) Cluster analyses of the control group and the tramadol group (blue represents dysregulated, gray means that there is no distinguished difference between the tramadol group and the control group); (**D**) Volcano plots and heatmaps of proteins with differential expression (FC > 1.2or FC < 0.833, P value < 0.05).

Information on proteome data was derived as follows: number of peptide-spectral matches (i.e., several spectra hits for the same peptide), unique peptide number, protein groups, and quantified proteins, which were 13,776, 2557, 469, and 443, respectively. All quantified proteins were shown in Table 1S. A boxplot of normalized density is shown in Fig. 1A. In addition, pairwise Pearson’s correlation coefficients from all six samples (3 replicates × 2 groups) were used to assess the relative quantitative reproducibility of the proteins and the results showed high reproducibility (R > 0.94) (Fig. 1B).

Total of 31 differential expressed proteins (DEPs) were found, of which nine proteins were up-regulated and 22 proteins were down-regulated, as shown in Fig. 1C,D and Table 1.

**Table 1:** The differential expression proteins in H vs N.

Identified protein names	Protein IDs	Score	FC	P	Molecular function
Glyceraldehyde-3-phosphate dehydrogenase	Q8CEV2	13.161	1.873294763	0.03772571	Oxidative reductase activity
Apolipoprotein C-III	A0A0R4J1N3	323.31	1.305433615	0.01459075	Enzyme inhibitor activity
Retinol-binding protein 4	H7BWY6	323.31	1.202682984	0.00012348	Retinol transporter activity; lipid transporter activity; lipid binding; retinol binding
Transthyretin	Q5M9K1	323.31	1.470107619	0.00626931	Thyroid hormone-binding protein
Alpha-1-antitrypsin 1-2	P22599	142	1.209979	0.00485745	Enzyme inhibitor activity; peptidase inhibitor activity
Tropomyosin alpha-1 chain	Q8BSH3	26.514	1.260379028	0.03965987	Structural constituent of cytoskeleton
Apolipoprotein C-II	Q3UJG0	7.7292	1.364705953	0.00107894	Enzyme activator activity; enzyme regulator activity; molecular function regulator
Apolipoprotein M	Q9Z1R3	94.515	1.237485713	0.00206508	Antioxidant activity; anion binding
Tubulin alpha-4A chain	A0A0A0MQA5	7.6679	0.711702291	0.01147102	Structural constituent of cytoskeleton
14-3-3 protein zeta/delta	A0A2I3BQ03	33.122	0.637214709	0.02060388	Cadherin binding; enzyme binding; ubiquitin protein ligase binding; cell adhesion molecule binding; protein kinase binding
Cofilin-1; Cofilin-2	Q544Y7	24.855	0.801105533	0.03488061	Actin filament binding; cytoskeletal protein binding
Major urinary protein 6; major urinary proteins 11 and 8	A2CEK7	20.781	0.710121107	0.03493991	Transporter activity; transmembrane receptor protein tyrosine kinase activity; kinase activity
Actin, cytoplasmic 1	Q3UAF7	217.53	0.805697691	0.00338196	Anion binding; carbohydrate derivative binding; purine ribonucleoside triphosphate binding; ATP binding
Filamin-A	B7FAV1	90.22	0.783548733	0.02197410	Actin filament binding; actin binding; cytoskeletal protein binding
Peptidase inhibitor 16	E9QNE5	115.97	0.8264383	0.01615836	Enzyme inhibitor activity; peptidase inhibitor activity
Calmodulin-like protein 3	P0DP28	217.53	0.583894819	0.00388330	Enzyme binding; phosphatidylinositol 3-kinase binding; glutamate receptor binding; calcium-dependent protein binding; G-protein coupled receptor binding
Talin-1	Q3UHS6	54.399	0.734627417	0.00545763	Actin filament binding; structural constituent of cytoskeleton; cytoskeletal protein binding
Plasma protease C1 inhibitor	P97290	103.99	0.807485012	0.00194138	Enzyme inhibitor activity; peptidase inhibitor activity
Haptoglobin	Q3UBS3	110.08	0.35444712	0.01797707	Hemoglobin binding; serine-type endopeptidase activity
Inter-alpha-trypsin inhibitor heavy chain H3	Q61704	183.26	0.806781264	0.03295815	Enzyme inhibitor activity; peptidase inhibitor activity
Vinculin	Q64727	323.31	0.769615002	0.01528594	Actin filament binding; cytoskeletal protein binding
Tropomyosin alpha-4 chain	Q6IRU2	45.874	0.680481412	0.01468016	Actin filament binding; cytoskeletal protein binding
Hemopexin	Q91X72	323.31	0.756880662	0.00440384	Heme transporter activity; cofactor transporter activity; organic cyclic compound binding
Carboxypeptidase B2	Q9JHH6	44.331	0.806708298	0.01316768	Zine ion binding; metallopeptidase activity; hydrolase activity

**Figure 2:**
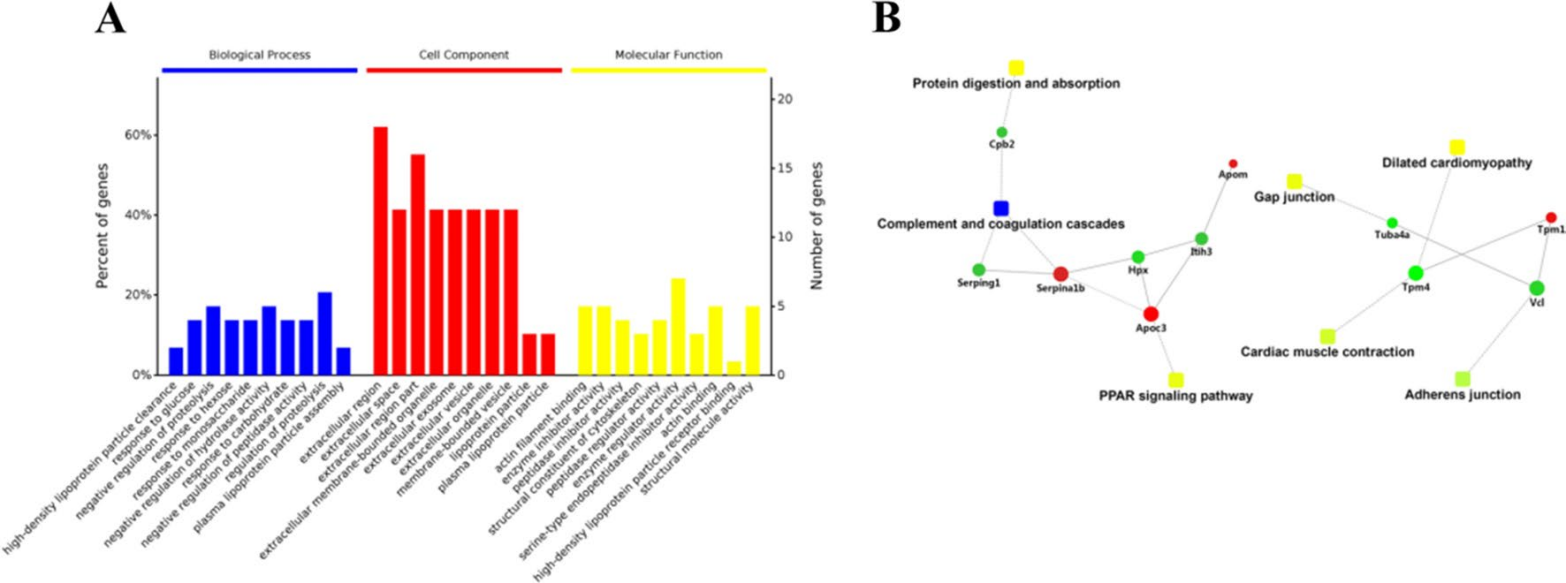
(**A**) Gene ontology (GO) term (including biological process, cellular component, and molecular function) enrichment for differential expressed proteins in the tramadol group and the control group. (**B**) Protein–protein interaction (PPI) networks of differential expressed proteins (DEPs) between the H and N group. Red nodes indicate up-regulated proteins and green nodes indicate down-regulated proteins. The rectangular node represents the KEGG pathway/biological process. The P-value is represented by a yellow-blue color gradient; the yellow color indicates a small P-value and the blue color indicates a large P-value.

All DEPs were classified into different Gene Ontology (GO) terms, biological process (BP), cell component (CC) and molecular function (MF) (Fig. 2A).

The top GO terms for BP enriched by DEPs in H group vs. N group were the regulation of proteolysis. The prominent GO CC categories that were enriched by these proteins included the extracellular region. The DEPs were associated with structural constituents of the cytoskeleton, actin filament binding, enzyme regulator activity, and structural molecule activity. In the PPI network of the DEPs, proteins such as Cpb2 (down-regulated), HpX (down-regulated), Itih3 (down-regulated), Serpina1b (up-regulated), serping1(down-regulated), Apom (up-regulated), Tuba4a (down-regulated), Vcl (down-regulated) and ApocIII (up-regulated) were mainly take part in the following KEGG pathways: protein digestion and absorption, the peroxisome proliferator-activated receptor (PPAR) signaling pathway, cardiac muscle contraction and adherens junctions (Fig. 2).

Some feature of DEPs’ changes were similar to other toxicological studies of the substance subject to abuse (Table 2)^10,17,28–33^.

**Table 2:** Summary of proteins modified by tramadol and one or more other substances of abuse.

Protein	Evidence from other drug researches
**Enzyme inhibitor-associated proteins**	
Apolipoprotein C-III (Apoc-III)	Alcohol^28^
Apolipoprotein CII (Apoc-II)	Alcohol^28^
Inter-alpha-trypsin inhibitor heavy chain H3 (itih3)	Alcohol^29^
**Mitochondria-related proteins**	
14-3-3 protein zeta/delta (YWHAZ)	Tramadol^10^
**Cytoskeleton proteins**	
Vinculin (Vcl)	Nictoine^30,31^
Hemopexin (Hpx)	Nicotine^32^
**Others**	
Haptoglobin (Hp)	Amphetamine^33^, heroin^17^
Transthyretin (Ttr)	Alcohol^29^, heroin^17^
Retinol-binding protein 4 (RBP-4)	Alcohol^29^

**Figure 3:**
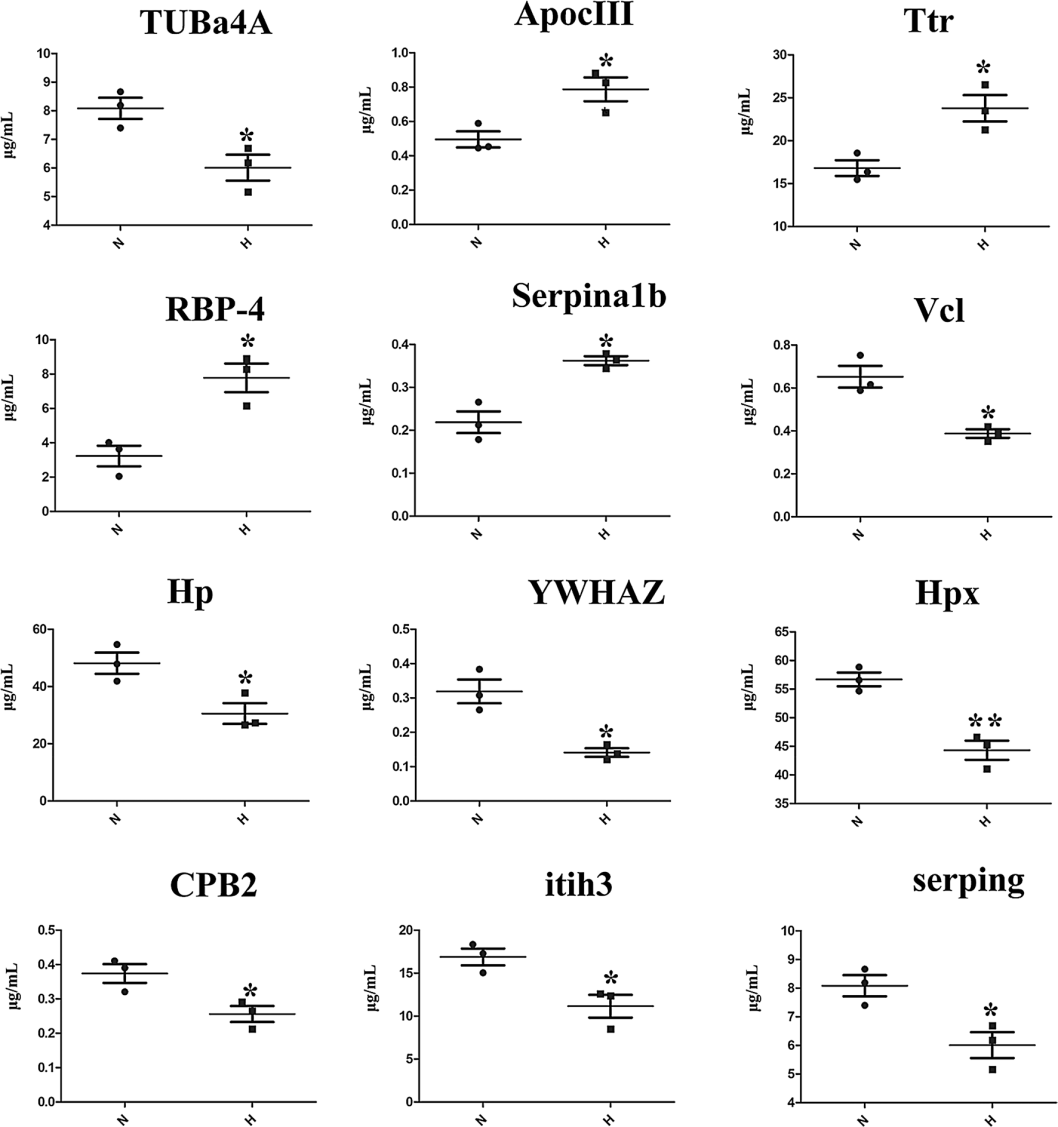
Elisa analyses of 12 differential expressed proteins. Changes in protein abundance shown by Elisa analysis and quantification of the proteins were highly consistent with the proteomic data of mice in serum. *P < 0.05, **P < 0.01.

While the expression level of ApocIII, Ttr, RBP-4 and TUBa4A was up-regulated, the expression level of Serpina1b, Vcl, Hp, YWHAZ, Hpx, CPB2, Itih3 and serping were down-regulated by Elisa (Fig. 3).

### Metabolomics results

The results of QC control data showed that the response strength and retention time of each chromatographic peak overlapped basically. The Pearson’s correlation coefficient among QC samples was calculated based on the peak area value and showed that the variation caused by instrument error is small and the data quality is reliable (Supplementary Fig. 3).

**Figure 4:**
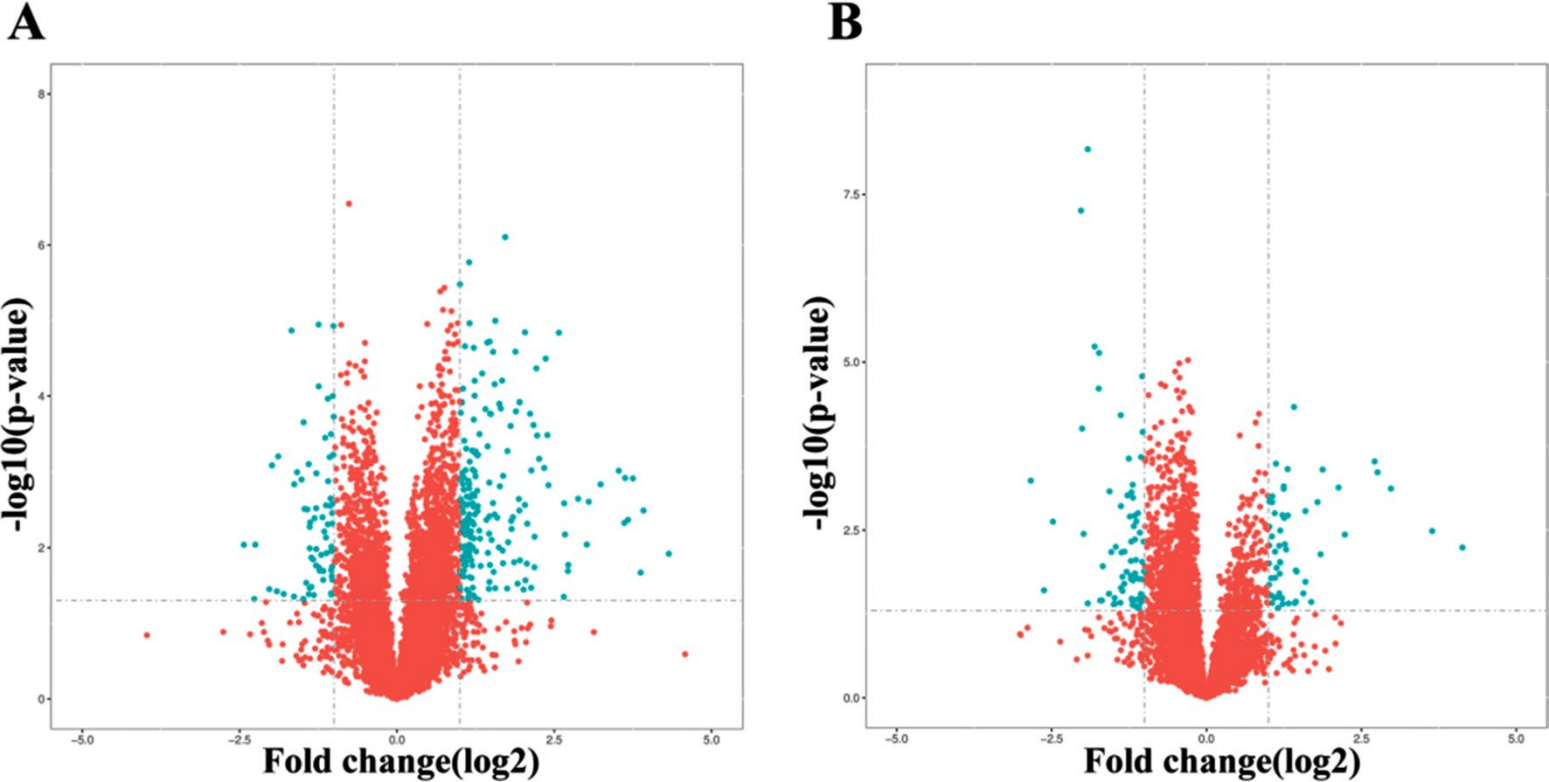
Volcano plots of metabolites with differential expression between the tramadol group and the control group (the (**A**) was obtained under negative ion mode; the (**B**) was obtained under positive ion mode, blue represents the distinguished metabolites (FC > 2 or FC < 0.5 and P value < 0.05, P value is calculated by t-test formula).

There are 29 differential expressed metabolites (DEMs) between the H and N group (Fig. 4 and Table 3).

**Table 3:** The distinguished different metabolites in H vs N groups.

No.	Metabolite	VIP	FC (H/N)	P value	Trend	Metabolic pathway
1	1-Methylnicotinamide	1.43537	2.661641219	4.62E − 05	↑	Nicotinate and nicotinamide metabolism
2	l-Valine	2.07855	1.599308079	0.000135188	↑	Protein digestion and absorption; Valine, leucine and isoleucine biosynthesis
3	l-Leucine	1.98634	1.721599218	0.00056504	↑	Protein digestion and absorption; Valine, leucine and isoleucine biosynthesis
4	Acacetin	1.31842	4.382382741	0.000732112	↑	Flavone and flavonol biosynthesis
5	Isoleucine	4.60723	2.37196399	0.000764949	↑	Protein digestion and absorption; Valine, leucine and isoleucine biosynthesis
6	l-Carnosine	1.40957	0.439065869	0.001036839	↓	Histidine metabolism
7	Cholesterol sulfate	2.09954	0.67083571	0.001286897	↓	Steroid hormone biosynthesis
8	Corticosterone	4.11877	0.517566612	0.001338819	↓	Steroid hormone biosynthesis
9	N-Cinnamoylglycine	4.15159	3.106462903	0.001544527	↑	The metabolites of glycine
10	Nicotinamide	3.96743	2.338651712	0.00188947	↑	Nicotinate and nicotinamide metabolism; Vitamin digestion and absorption
11	Benzoic acid	1.80976	1.385671507	0.002237064	↑	Degradation of aromatic compounds
12	20-Hydroxyeicosatetraenoic acid	7.79954	0.715070263	0.002618757	↓	Vascular smooth muscle contraction
13	Kynurenic acid	2.21865	2.074864706	0.003409024	↑	The catabolites of tryptophan
14	Phenylacetylglycine	4.07605	1.973829943	0.004025549	↑	Phenylalanine metabolism
15	Mesaconic acid	2.02296	0.763354166	0.0055795	↓	C5-Branched dibasic acid metabolism
16	dl-Beta-hydroxybutyric acid	6.80608	0.621292252	0.006004963	↓	Synthesis and degradation of ketone bodies
17	l-Norleucine	1.7419	2.275386538	0.009433052	↑	Protein digestion and absorption; Valine, leucine and isoleucine biosynthesis
18	Creatine	1.08606	1.333084778	0.009437955	↑	Arginine and proline metabolism; Glycine, serine and threonine metabolism
19	l-Phenylalanine	1.77148	1.434736131	0.010075828	↑	Protein digestion and absorption; Phenylalanine metabolism
20	Taurochenodeoxycholate	2.53522	0.434162682	0.012439044	↓	Bile secretion; Secondary bile acid biosynthesis; Primary bile acid biosynthesis
21	Alpha-chaconine	1.92791	0.771722033	0.015423328	↓	Glycerophospholipid metabolism
22	Pantothenate	1.3127	1.487048436	0.015648029	↑	Vitamin digestion and absorption; Pantothenate and CoA biosynthesis
23	Eicosapentaenoic acid	3.8208	1.271415181	0.019745226	↑	Fatty acid biosynthesis
24	l-Pipecolic acid	1.40422	0.6239531	0.022905318	↓	Lysine degradation
25	Glutamine	1.93396	1.300065997	0.023490107	↑	GABAergic synapse; d-glutamine and d-glutamate metabolism; Glutamatergic synapse
26	Cytidine	1.49422	1.168843272	0.023943668	↑	Pyrimidine metabolism
27	2-Hydroxy-4-methylpentanoate	5.51063	1.960514129	0.025465597	↑	Organic acid
28	Indoleacrylic acid	1.10864	1.885752926	0.03537406	↑	The metabolites of tryptophan
29	Dodecanoic acid	2.28455	0.628753946	0.049332248	↓	Fatty acid biosynthesis

**Figure 5:**
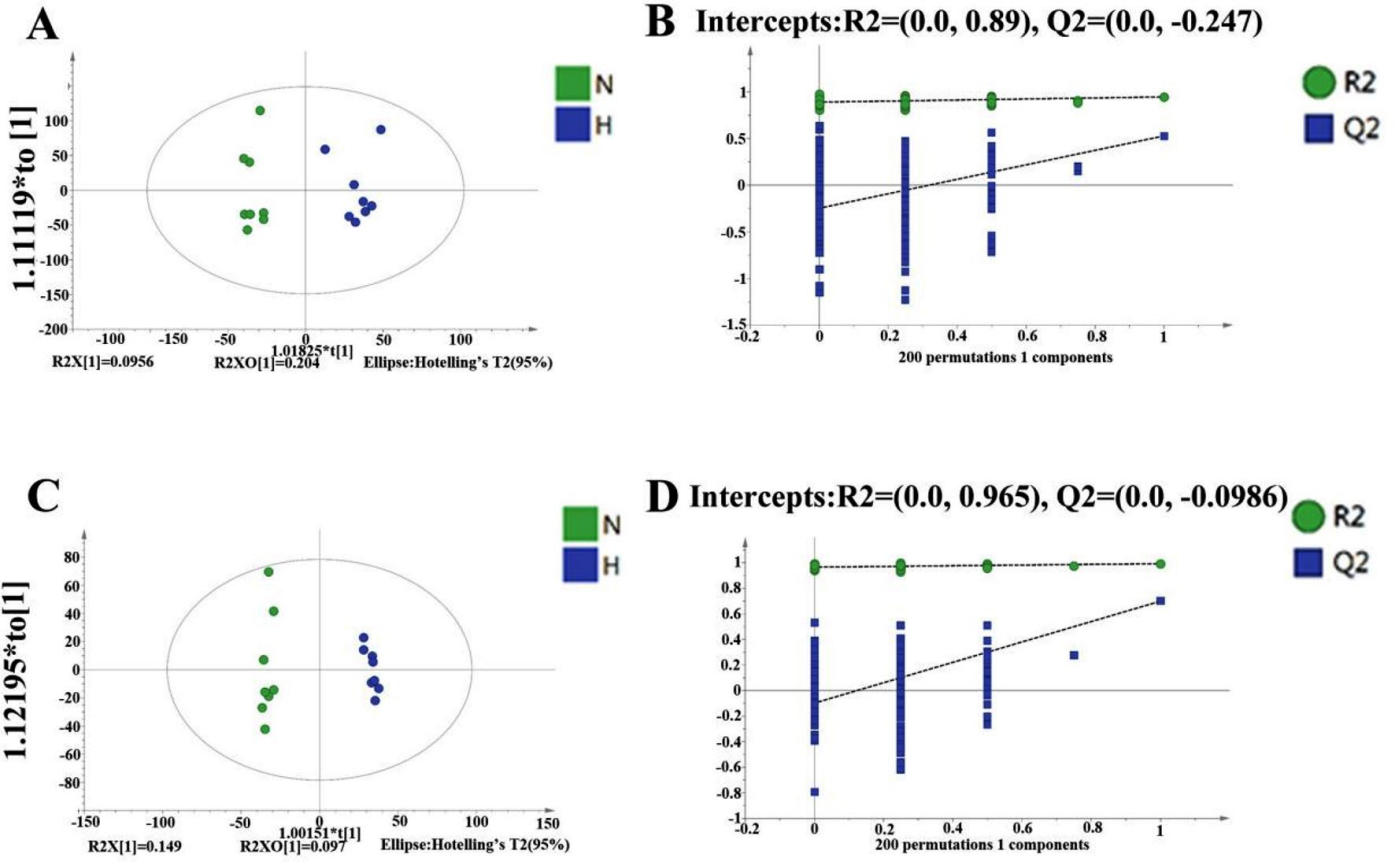
OPLS-DA Score Results and OPLS-DA valid figure of Mouse Serum Samples (**A**, **C**) represent the OPLS-DA score results; (**A**) was obtained under negative ion mode; (**C**) was obtained under positive ion mode; (**C**, **D**) represent the OPLS-DA valid, (**B**) was obtained under negative ion mode, (**D**) was obtained under positive ion mode; intercepts: R^2^ and Q^2^ represent y-intercept of R^2^ and Q^2^ regression lines. N represents the control group; H represents the tramadol group.

The PCA results of all groups did not show a satisfactory separation of data between the tramadol exposure group and the control group, that was shown in Supplementary Fig. 4. The variance (R^2^) and model predictability (Q^2^) for the OPLS-DA were calculated to be 0.992 and 0.699 under negative mode, and R^2^ and Q^2^ for the OPLS-DA were calculated to be 0.947 and 0.529 under positive mode. In conclusion, the model had good reliability and predictability as shown in Fig. 5.

**Figure 6:**
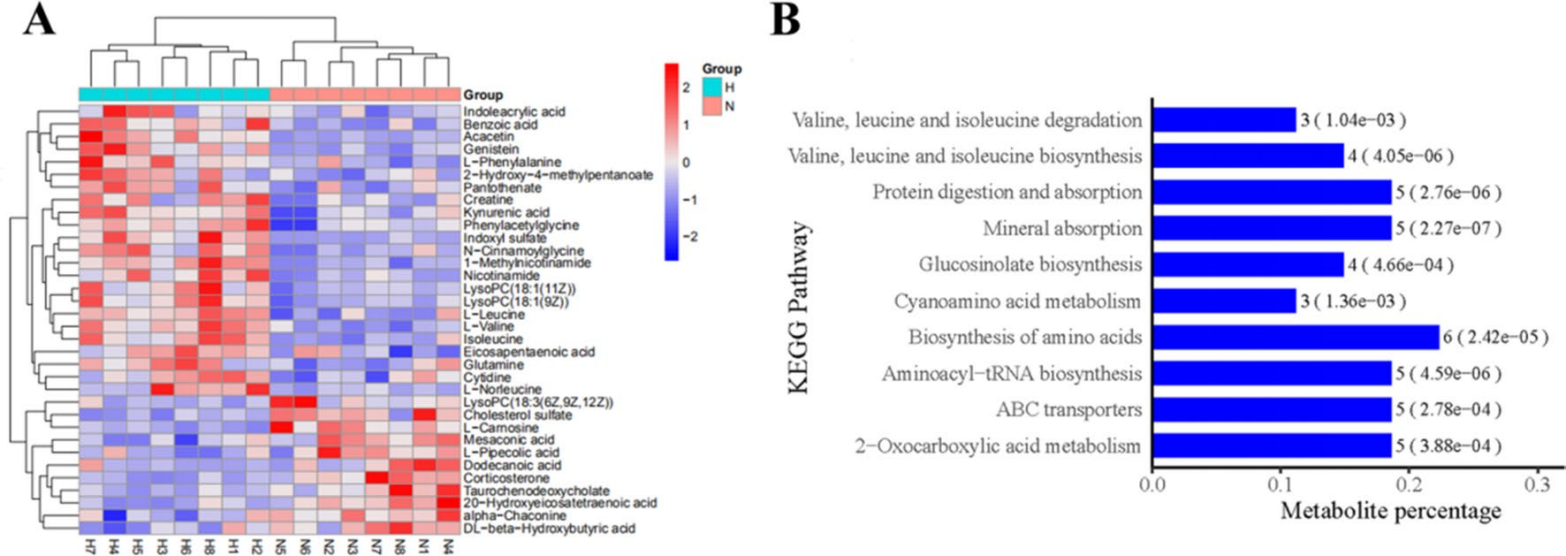
(**A**) Cluster analysis of the tramadol group and the control group (red represents up-regulated, blue represents down-regulated). (**B**) Enrichment KEGG pathway (top 10 between the tramadol group and the control group, H represents the tramadol group; N represents the control group).

The metabolomic profile of the H and N group is different (Fig. 6A).These DEMs were enriched for the KEGG pathway “biosynthesis of amino acids”, “protein digestion and absorption”, “valine, leucine and isoleucine biosynthesis” and “valine, leucine and isoleucine degradation” etc. (Fig. 6B).

### Integrated analysis of metabolomics and proteomics

**Figure 7:**
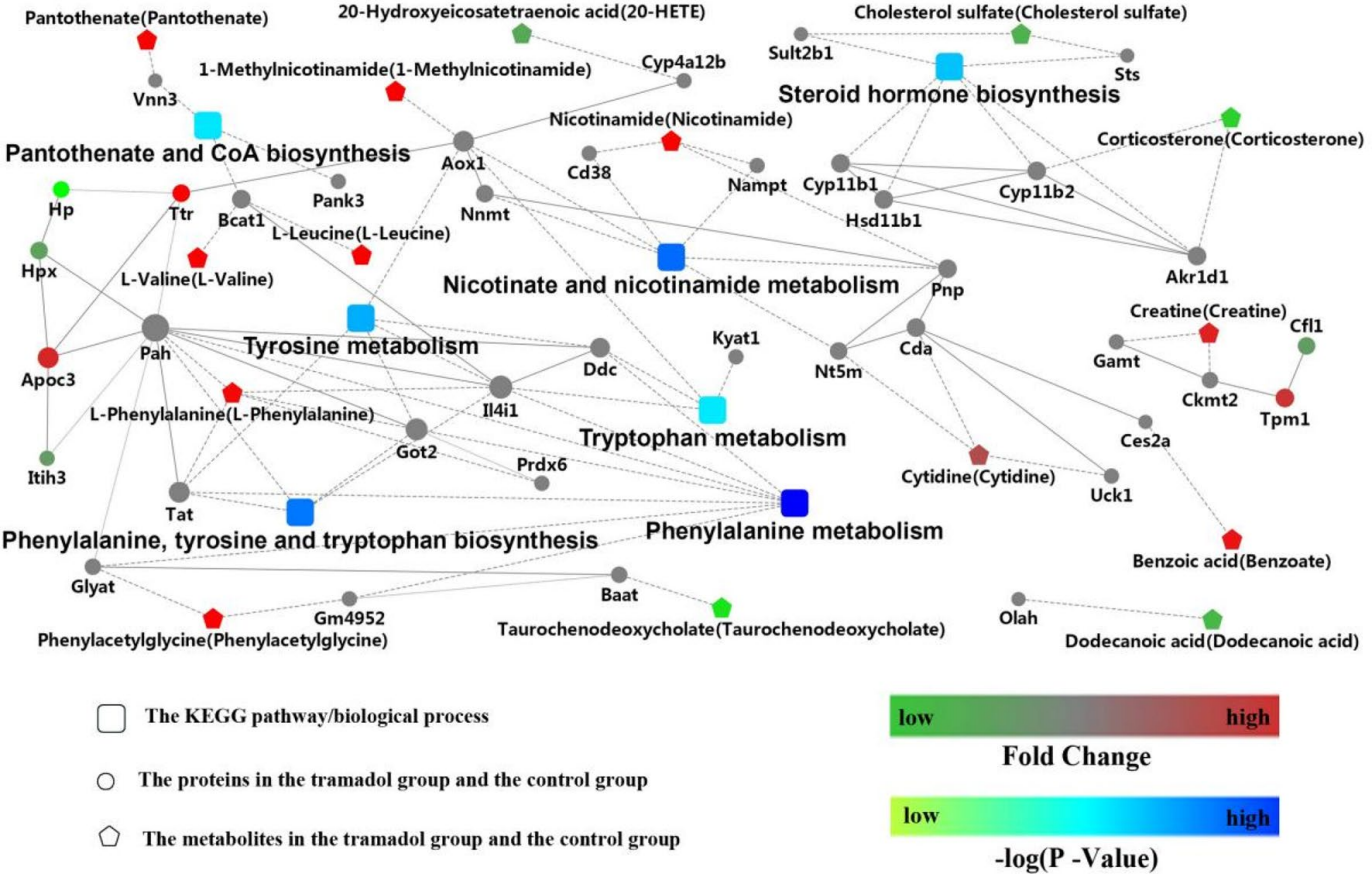
Combination analyses with proteomics and metabolomics. Red represents up-regulated and green represents down-regulated. The rectangular node represents the KEGG pathway/biological process. The circular and pentagon nodes represent the proteins and the metabolites in the tramadol group and the control group, respectively. The P-value is represented by a blue color gradient; the deeper blue color indicates a larger P-value.

The DEPs and DEMs in the H and N group mainly were involved in nicotinate and nicotinamide metabolism, phenylalanine metabolism and the PPAR signaling pathway as shown in Fig. 7. From the Fig. 2S, these DEMs and DEPs were enriched for the KEGG pathway “biosynthesis of amino acids”, “steroid hormone biosynthesis”, “phenyalaine metabolism”, “tyrosine metabolism”, “nicotinate and nicotinamide metabolism”, “focal adhesion”. Moreover, the common enrichment KEGG pathway for DEPs and DEMs is “protein digestion and absorption” including the upregulation of l-isoleucine; l-valine; l-leucine; l-phenylalanine; l-glutamine and down-regulated Cpb2 by integrated analysis of metabolomics and proteomics.

## Discussion

Although tramadol has become the most prescribed opioid worldwide, there are few assays validated the effect of chronic exposure to tramadol. To obtain the comprehensive system biology profile, two ‘omics’ pipelines utilizing LC–MS/MS instrumentation were developed and leveraged for this work. We found that 31 DEPs and 34 DEMs in the H and N groups. Moreover, KEGG pathway “protein digestion and absorption” was the key pathway in the effects of tramadol.

Given the serum is obtained easily in the clinic practice, and few reports focused on the effects of tramadol on proteins and metabolites in serum, so we chose the serum as the sample. In the present study, TMT-LC–MS/MS-based quantitative proteome analysis showed protein changes in the metabolic process between the H and N groups. By bioinformatics analysis,we found that these proteins were classified into different Gene Ontology (GO) terms, biological process (BP), cell component (CC) and molecular function (MF) (Fig. 2A). The top GO terms for BP enriched by DEPs in H (the tramadol group) vs. N (the control) group were the regulation of proteolysis. The prominent GO CC categories that were enriched by these proteins included the extra-cellular region. Bioinformatics analyses also indicated that these DEPs were associated with structural constituents of the cytoskeleton, actin filament binding, enzyme regulator activity, and structural molecule activity.

To further investigate the functions of these DEPs, protein–protein interaction (PPI) networks were constructed for proteins with differential expression in the H and N group. As shown in Fig. 2B, these DEPs mainly take part in the following KEGG pathways: protein digestion and absorption, the peroxisome proliferator-activated receptor (PPAR) signaling pathway, cardiac muscle contraction and adherens junctions. The DEPs in the PPI networks had a relatively high degree of connectivity, what’s more, we validated these differential proteins by Elisa kit. The effect of long term exposure to tramadol was associate with protein digestion and absorption, the PPAR signaling pathway, cardiac muscle contraction and adherens junctions.

By comparing our experimental results with similar toxicological studies of the substance subject to abuse, we found similar DEPs shown in Table 3. Some DEPs (Apoc III, Apoc II, itih3, YWHAZ, Vcl, Hpx, Hp, Ttr, RBP-4) in our study were compared with those differential proteins induced by other addiction drug i.g. nicotine, alcohol, morphine, heroin, amphetamine, and tramadol in Table 3. Actin and Vcl were also down-regulated after nicotine abuse. The down-regulated Hp and up-regulated Ttr were also reported after alcohol and heroin abuse, what’s more, variation trend of Hp and Ttr induced by tramadol was coordinate with the heroin addicts. Hp is a highly abundant plasma glycoprotein, its main function is to combine with free haemoglobin (Hb) to form stable Hp–Hb complexes. The bioinformatics analysis of Hp showed that Hp is important in the molecular regulation of inflammation, which also acts as an antioxidant, has antibacterial activity and plays a role in modulating many aspects of the acute phase response^34–36^. The up-regulated RBP-4 was also found in the heroin addicts, suggested it may be a potential bio-marker for opioid abuse. The down-regulated Hpx was observed in our study, and Cecconi D reported similar results induced by nicotine abuse^30^. Hpx is a plasma protein belonging to positive acute-phase proteins that binds and transports haeme, thus preventing oxidative damage^32^. All the reports mentioned above, integrated with our findings, suggesting tramadol and these drugs caused similar changes in cellular activities and biological processes in the serum.

In order to obtain comprehensive profile change after exposure to tramadol, the changes in the metabolome were also be investigated. The metabolomic pattern was distinguished differences between the H and N groups. Creatine was suggested be correlated with kidney injury^37^. 1-Methylnicotinamide and nicotinamide are involved in nicotinate and nicotinamide metabolism. Moreover, they were suggested to be correlated with inflammation and oxidative damage^3,26^. Tryptophan can be transformed into 5-HT, which can also be transformed into indole acrylic acid and kynurenic acid^38^. We speculated the balance between tryptophan and 5-HT is disrupted by the up-regulation of indole acrylic acid and kynurenic acid. The branched-chain amino acids (BCAC, including l-isoleucine, l-leucine and l-valine) can stimulate the proliferation of monocytes, to enhance immune response. Rebholz suggested that an imbalance of BCAC may lead to immune damage, that is correlated with inflammation to some extent^39^. In addition, hydroxybutyric acid, glutamine and phenylalanine were suggested to be a correlation with neurotransmitter disruption^6^. Taken together, the enrichment KEGG pathway showed that these different metabolites take part in the biosynthesis of amino acids, protein digestion and absorption, valine, leucine and isoleucine biosynthesis and valine, leucine and isoleucine degradation.

In this context, the multi-omics was used to evaluate the effect of exposure to tramadol. In Fig. 7, we can see that the Nicotinate and nicotinamide metabolism and phenylalanine, tyrosine and tryptophan metabolism were affected after exposure to tramadol, that is coordinate with the relevance report^2,4^. Combination analyses of proteomics and metabolomics showed that Cpb2 could be used to elucidate the effect of tramadol. Cpb2 is a basic carboxypeptidase which can attenuate fibrinolysis and plays a role in regulating complement activation in vivo^40,41^, also shows anti-inflammatory activity in the presence of thrombin in vitro^42^. Carboxypeptidase B (CpB) which cleaves carboxy-terminal lysine residues, abolished reactive oxygen species induced by oxidative stress^43^. The downregulation of Cpb2 was speculated to weaken the defense of organs during oxidative damage caused by long-term exposure to tramadol.

Limitation are exist in the study, First, the serum proteome coverage reported is sub-optimum and we just suggested that a few differential protein can be used as potential key proteins to elucidate the toxicity of chronic exposure to tramadol, further investigations need to be performed for the identification of their modification in proteins. Second, for the ethical purpose, only eight mice, which can meet the purpose of metabolomics analysis, were included in each group in the present study. In addition, quantitative studies of proteomics need to be performed to validate others DEPs not included in Fig. 3 after tramadol exposure in future research. As the high abundance proteins in serum were not removed, the low abundance proteins may not be detected, that result in the proteomics profile not the most comprehensive.

## Conclusion

In conclusion, the proteomic and metabolomic profiles were significantly changed over a 5 weeks following exposure to tramadol, several protein and metabolite markers have been found to be significantly changed. These DEPs can be linked to protein digestion and absorption, the PPAR signaling pathway, cardiac muscle contraction and adherens junctions. These DEMs were enriched for the KEGG pathway “biosynthesis of amino acids”, “protein digestion and absorption”. Integrated analyses of proteomics and metabolomics, the common KEGG pathway is protein digestion and absorption. Collectively, our findings may provide the fundamental data for toxicity of tramadol in serum.

## Supplementary Information

Supplementary Information 1.

Supplementary Figure 1.

Supplementary Figure 2.

Supplementary Figure 3.

Supplementary Figure 4.

Supplementary Table 1S.

## Data Availability

The mass spectrometry proteomics data have been deposited to the ProteomeXchange Consortium (http://proteomecentral.proteomexchange.org) via the iProX partner repository with the dataset identifier PXD019233. All data are fully available without restriction.
