# Supplementary material for: Changes on proteomic and metabolomic profile in serum of mice induced by chronic exposure to tramadol
Source: Sci Rep. 2021 Jan 14;11:1454. doi: 10.1038/s41598-021-81109-7 (PMC7809287; doi:10.1038/s41598-021-81109-7)
Supplement: Supplementary file 1 — Supplementary Information 1. [file 41598_2021_81109_MOESM1_ESM.zip › supplent file -method.docx]

**2.3.3 TMT peptide labeling, fractionation and LC-MS/MS analysis**

Peptides were labeled with TMT reagents based on the manufacturer’s instructions (Thermo Fisher Scientific).To quantify six samples, each aliquot (100 µg of peptide equivalent) was reacted with one tube of TMT reagent. After the sample was dissolved in 100 µL of 0.05 M tetraethylammonium bromide (TEAB) solution, pH 8.5, the TMT reagent was dissolved in 41 µL of anhydrous acetonitrile. The mixture was incubated at room temperature for 1 hour.Then, 8 µL of 5% hydroxylamine was added to the sample and incubated for 15 min to quench the reaction. The 6-plex labeled samples were pooled together and lyophilized. The schematic for the TMT-labeling strategy used for the samples as shown in Fig.1S.

The TMT-labeled peptides mixture was fractionated using a Waters XBridge BEH130 column (C18, 3.5 µm, 2.1 × 150 mm) on an Agilent 1290 high-performance liquid chromatography(HPLC) operating at 0.3 mL/min. Buffer A: 10mm ammonium formate, buffer B: 10mm ammonium formate was dissolved in 90% acetonitrile; Both buffers were adjusted to pH10 with ammonium hydroxide. A total of 30 fractions were collected for each peptide mixture and then concatenated to 15 (pooling equal interval RPLC fractions). The fractions were dried for nano LC-MS/MS analysis.

Separation was performed using an EASY-nLC1200 nanoflow HPLC system (Thermo Fisher Scientific, Karlsruhe, BW, Germany). The buffer used in the analysis included solvent A (0.1% formic acid (FA) in water) and solvent B (95% acetonitrile) with 0.1% FA. First, 100% solution A was used for equilibration of the column. The sample was loaded onto a Trap column (2 cm × 100 µm, 5 µm-C18) and transferred to a Thermo Scientifific EASY analytical column (75 µm × 120 mm, 3 µm-C18) at a 300 nL/min flow rate for separation. The relevant liquid phase gradient was as follows: 0–3 min, linear gradient from 2% to 7% buffer B; 3–48 min, linear gradient from 7% to 35% buffer B; 48–53 min, linear gradient from 35% to 90% buffer B; and 53–60 min, buffer B maintained at 90%. For LC/MS analysis, the peptides were subjected to tandem MS using a Q-Exactive Plus MS (Thermo Scientific). The parameters were as follows: detection method, positive mode; capillary voltage, 1.8 kV; isolation width, 1.6 Th; and parent ion scanning range recorded in the range of 300–1800 m/z. The mass-tocharge ratio of the fragments of the peptides and polypeptides was collected as follows: 20 fragment maps (MS2 scan, high energy collision dissociation (HCD)) were acquired after each full scan, which employed primary MS resolution of 70,000 (at m/z 200), AGC target values of 1e6, Level 1 maximum IT of 50 ms, and secondary MS resolution of 17,500 (at m/z 200), a target AGC value of 1e5, and Level 2 maximum IT of 50 ms (MS2 Activation Type: HCD; Isolation window: 2.0 Th; Normalized collision energy: 27).

**2.3.4 Database search and protein quantification**

The resulting LC-MS /MS original files were imported into the MaxQuant software program (version 1.6.1.0) for data interpretation and protein identification against the database uniprot-mus musculus-87573-20190710.fasta (released in July 2019 and including 87573 protein numbers), which was sourced from the protein database at https://www.uniprot.org/uniprot/?query=taxonomy:10090. An initial search was set at a precursor mass window of 4.5 ppm. The search follows the enzymatic cleavage rules of trypsin /P, allowing for a maximum of two missing cleavage sites and a mass tolerance of 20ppm for fragment ions.The modification set was as follows: Fixed modification: carbamidomethyl (C), TMT6plex (K), TMT6plex (n-term); Variable modification: oxidation (M) and acetyl (protein n). The minimum 7 amino acids for peptide, no less than 1 unique peptide was required per protein.

False discovery rate (FDR) for peptide and protein identification was set to 1%. The TMT reporter ion intensity was applied for quantification. The relative quantitative protein analysis of samples was performed using MaxQuant algorithms (http://www.maxquant.org, VERSION 1.6.0.16) (18).The mass spectrometry proteomics data have been deposited to the ProteomeXchange Consortium (http://proteomecentral.proteomexchange.org) via the iProX partner repository with the dataset identifier PXD019233(19). ‍

**2.4.2 Sample analysis and data preprocessing**

During the whole analysis process, the sample was placed in a 4℃ automatic sampler, and the sample was adopted Agilent 1290 Infinity LC. A HILIC column was used for separation in an ultra high performance liquid chromatography (UHPLC) system. The sample amount is 10 μl. The column temperature was 25℃ and the flow rate was 0.3 mL/min. Chromatographic mobile phase A: water +25 mM ammonium acetate +25 mM ammonia,B: acetonitrile; The chromatographic gradient elution procedure was as follows: 0-0.5 min, 95% B; 0.5-7 min, linear gradient from 95% to 65% solution B; 7-9 min, linear gradient from 65% to 40% solution B; 9-10 min, B maintained at 40%; 10-11.1min, B changes linearly from 40% to 95%; 11.1-16 min, B maintained at 95%.QC samples were used for monitoring and evaluation the stability of the liquid chromatography-mass spectrometry instrumental system and the availability of experimental data.

Positive and negative ion patterns were measured by electrospray ionization (ESI). Sample was analyzed by Triple TOF 5600 mass spectrometer (AB SCIEX) after UPLC separation. The ESI source Conditions are as follows: Ion Source Gas1 (Gas1) : 60, Ion Source Gas2 (Gas2) : 60, Curtain Gas (CUR) : 30, source temperature: 600℃, IonSapary Voltage Floating (ISVF) ±5500 V (plus or minus two modes); TOF MS scan m/z range: 60-1200 Da, product ion scan M /z range: 25-1200 Da, TOF MS scan accumulation time 0.15s /spectra, product ion Scan accumulation time 0.03s /spectra; Information dependent was adopted for mass spectrometry at the second level Acquisition (IDA) is acquired, and adopts high sensitivity mode, Declustering potential (DP) : ±60 V (plus or minus two modes), Collision Energy: 30 eV, IDA set as follows Exclude Isotopes within 4 Da, Candidate ions to monitor per cycle: 6, and dynamic exclusion time was 10 s.

The original data was transformed into. MzXML format by ProteoWizard, and then peak matching was carried out by XCMS program Alignment, retention time correction and extraction peak area.Parameter settings for XCMS processing were as follows: centWave for feature detection (Δ*m*/*z* = 5, minimum peak width = 5 s, and maximum peak width = 20 s); obiwarp settings for retention time correction (profStep = 1); parameters for chromatogram alignment included mzwid = 0.025, minfrac = 0.5, and bw = 5. Accurate mass number matching was used for metabolite structure identification (<25 PPM). The method of matching with the secondary spectrogram was firstly used to retrieve the self-built database of the laboratory.Secondly, Combined with the high-quality mzCloud database constructed by the standard sample and the mzVault and MassList databases, the molecular characteristic peaks were matched and identified, as many metabolites in the biological system as possible could be identified, and the information of the total metabolites was reflected to the greatest extent.The original data was preprocessed using CD data processing software for data search. First, the data was simply filtered and peak aligned by retention time and mass-to-charge ratio. The exact molecular weight of the compound was then determined by the mass-to-charge ratio in the high-resolution XIC diagram. According to the mass deviation and adduct ion information, the molecular formula was predicted. By matching the fragment ion, collision energy and other information of each compound in the mzCloud database, the metabolite in the biological system was identified. All the identified metabolites were identified by secondary mass spectrometry. By manual screening, only the metabolites with high matching degree and accurate identification were retained.Metabolites were annotated using the Kyoto Encyclopedia of Genes and Genomes (KEGG) (<http://www.genome.jp/kegg/>). The resulting matrix was imported into SIMCA-P (version 13.0, Umetrics, Sweden) for unsupervised principal component analysis (PCA) and orthogonal partial least square discriminant analysis (OPLS-DA)after mean centering and unit variance scaling. We applied univariate analysis (t-test) to calculate statistical significance (P-value). Metabolites with variable importance in the project (VIP) > 1, P-value < 0.05 were considered differential metabolites. Metabolites of interest were filtered based on values of VIP, Log2(FC), and -log10(P-value). Volcano plots were utilized to illustrate the distribution of differential metabolites.For clustering heat maps, data were normalized using z-scores of the intensity areas of differential metabolites and were plotted using the R package “pheatmap”.
