# Supplementary figures and images for: Changes on proteomic and metabolomic profile in serum of mice induced by chronic exposure to tramadol
Source: Sci Rep. 2021 Jan 14;11:1454. doi: 10.1038/s41598-021-81109-7 (PMC7809287; doi:10.1038/s41598-021-81109-7)

# 1-Methylnicotinamide

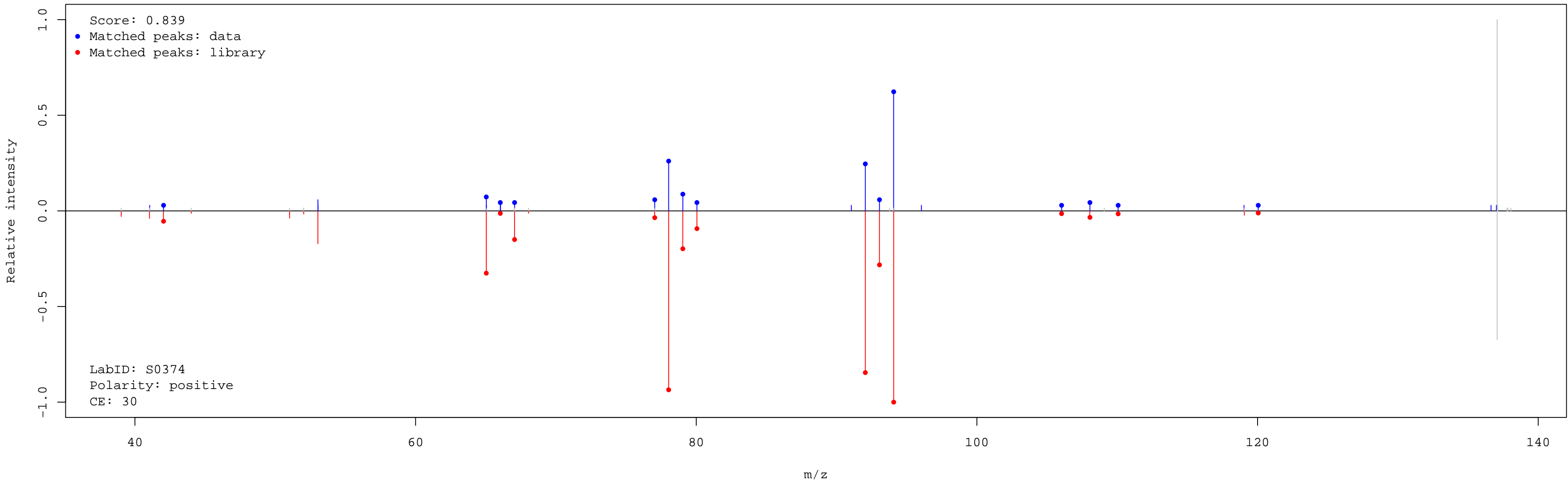

Supplement: Supplementary file 1 — Supplementary Information 1. [file 41598_2021_81109_MOESM1_ESM.zip › 0.839,1-Methylnicotinamide,M+.pdf]

20-Hydroxyeicosatetraenoic acid

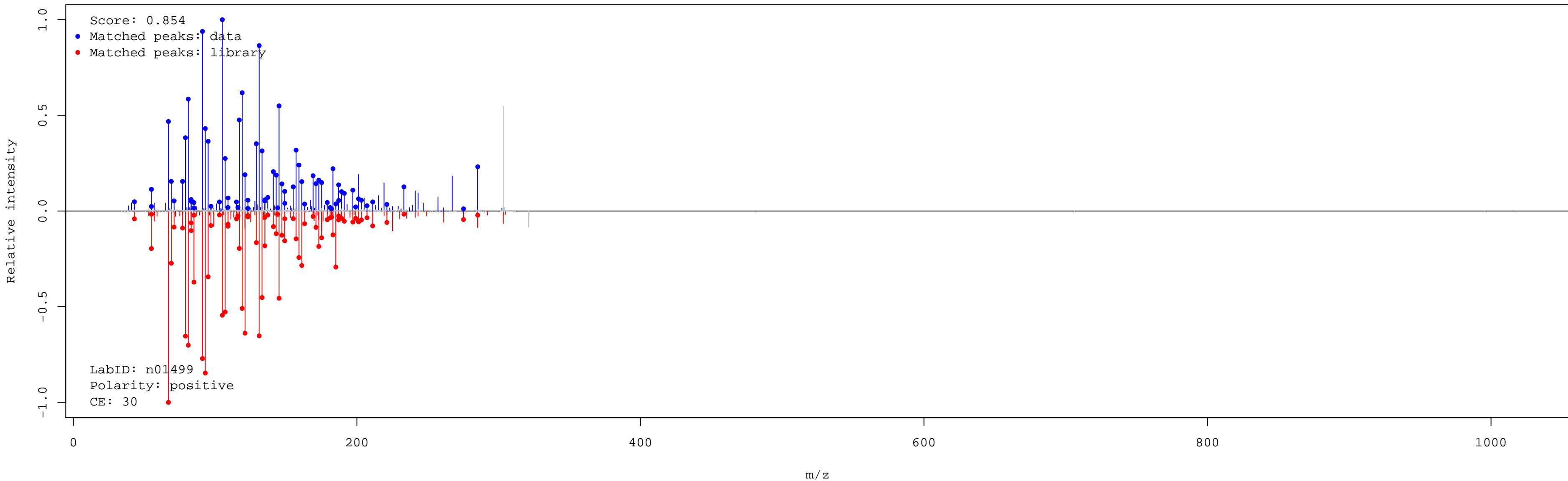

Supplement: Supplementary file 1 — Supplementary Information 1. [file 41598_2021_81109_MOESM1_ESM.zip › 0.854,20-Hydroxyeicosatetraenoic acid,(M+H-H2O)+.pdf]

Benzoic acid

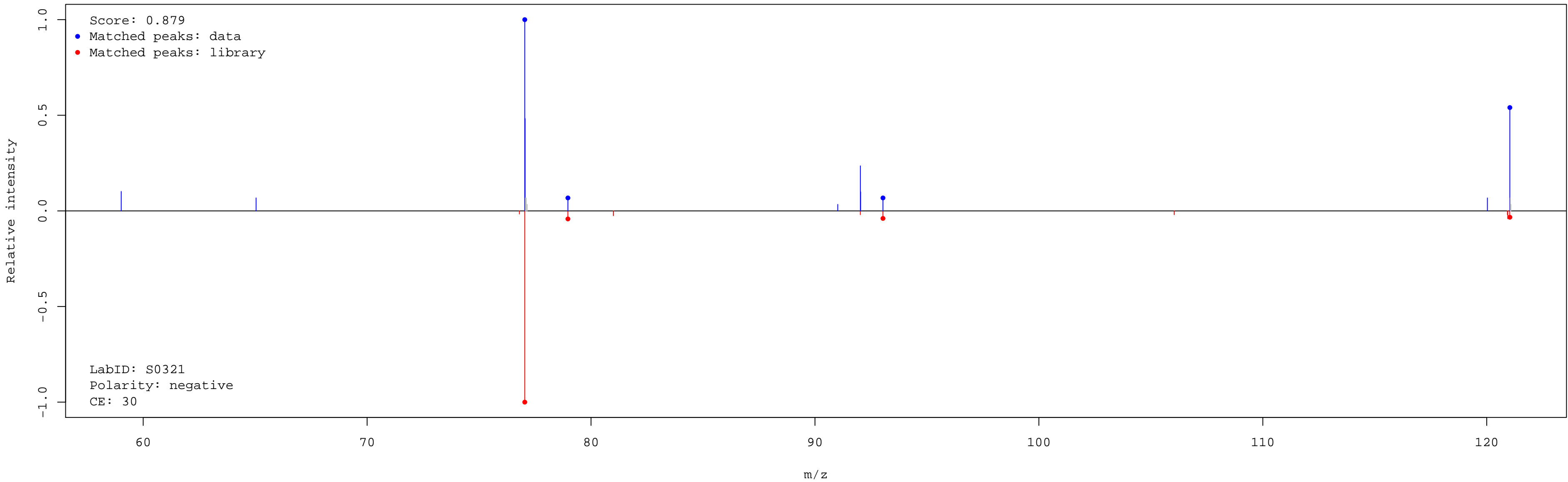

Supplement: Supplementary file 1 — Supplementary Information 1. [file 41598_2021_81109_MOESM1_ESM.zip › 0.879,Benzoic acid,(M-H)-.pdf]

# Cytidine

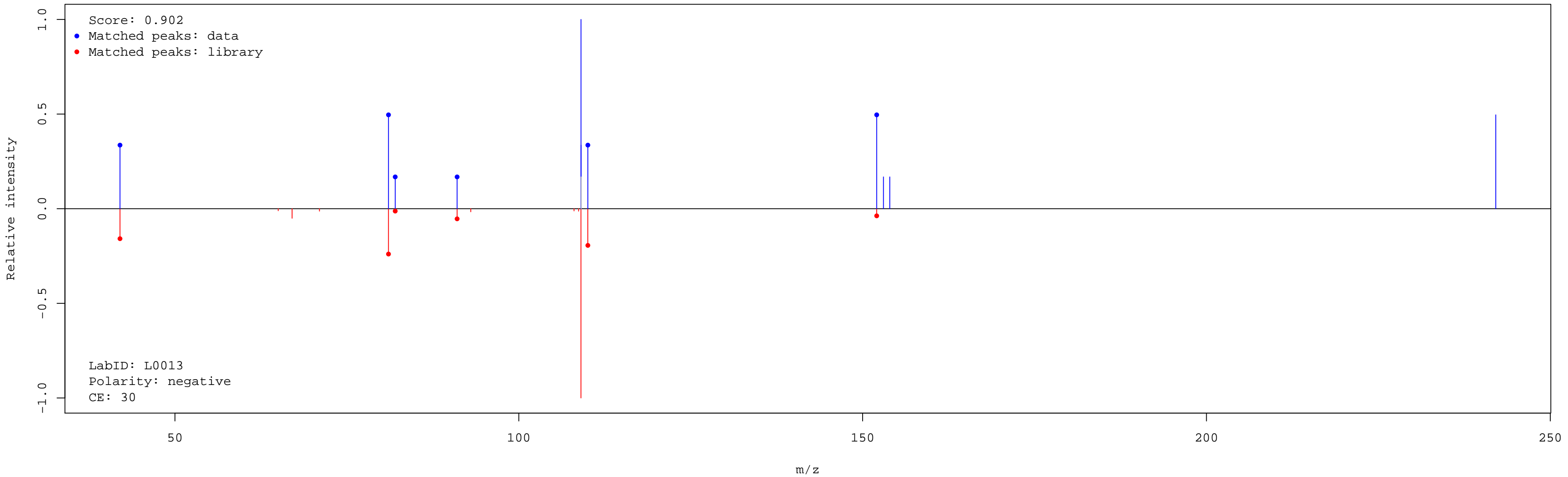

Supplement: Supplementary file 1 — Supplementary Information 1. [file 41598_2021_81109_MOESM1_ESM.zip › 0.902,Cytidine,(M-H)-.pdf]

# Eicosapentaenoic acid

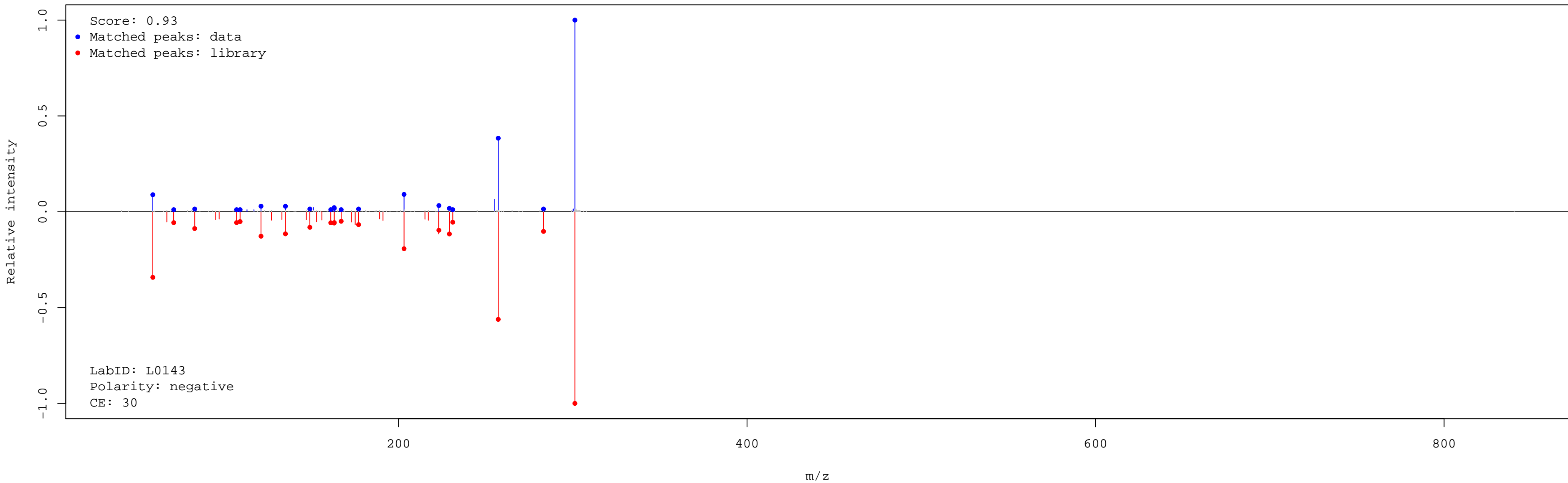

Supplement: Supplementary file 1 — Supplementary Information 1. [file 41598_2021_81109_MOESM1_ESM.zip › 0.93,Eicosapentaenoic acid,(M-H)-.pdf]

Pantothenate

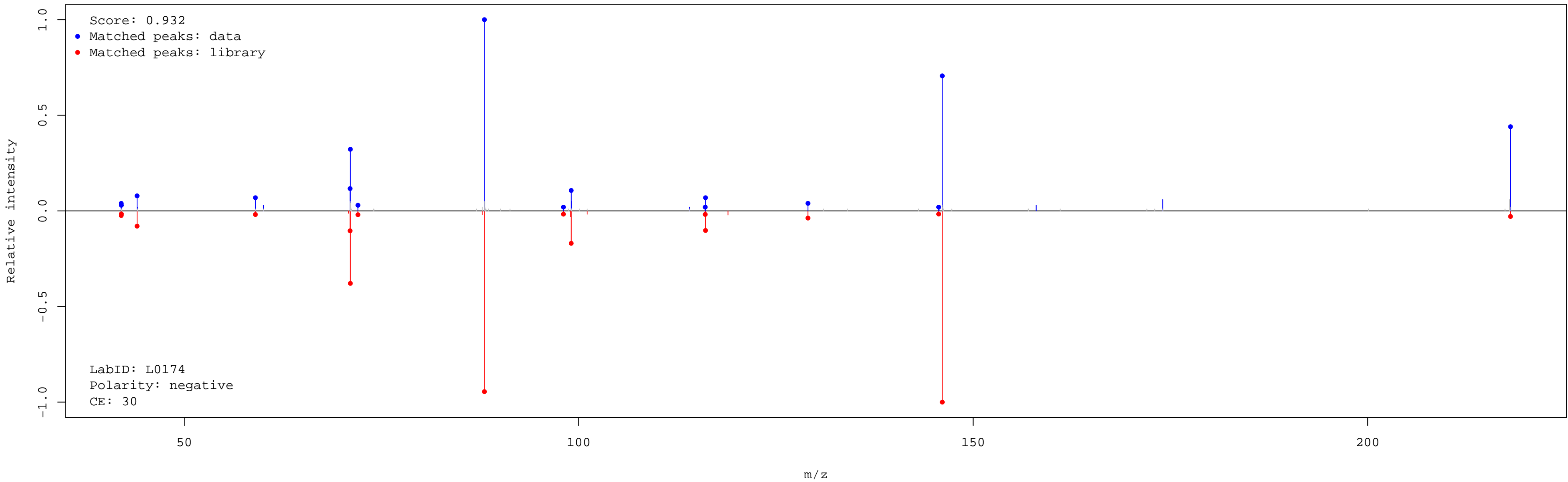

Supplement: Supplementary file 1 — Supplementary Information 1. [file 41598_2021_81109_MOESM1_ESM.zip › 0.932,Pantothenate,(M-H)-.pdf]

# L-Leucine

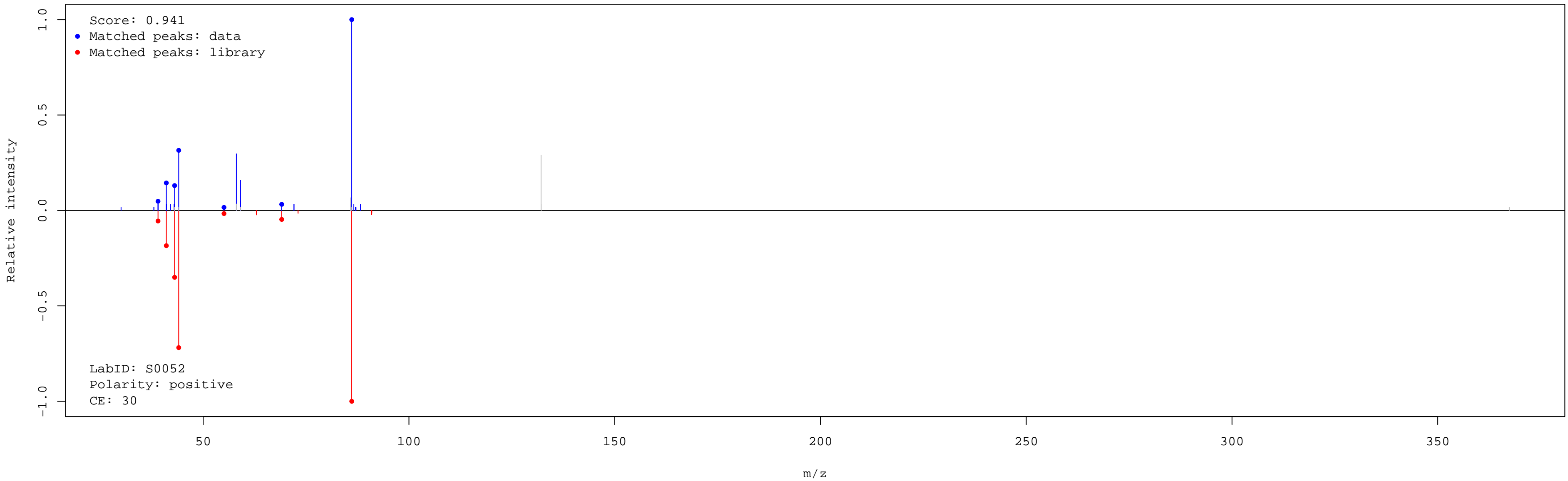

Supplement: Supplementary file 1 — Supplementary Information 1. [file 41598_2021_81109_MOESM1_ESM.zip › 0.941,L-Leucine,(M+H)+.pdf]

L-Norleucine

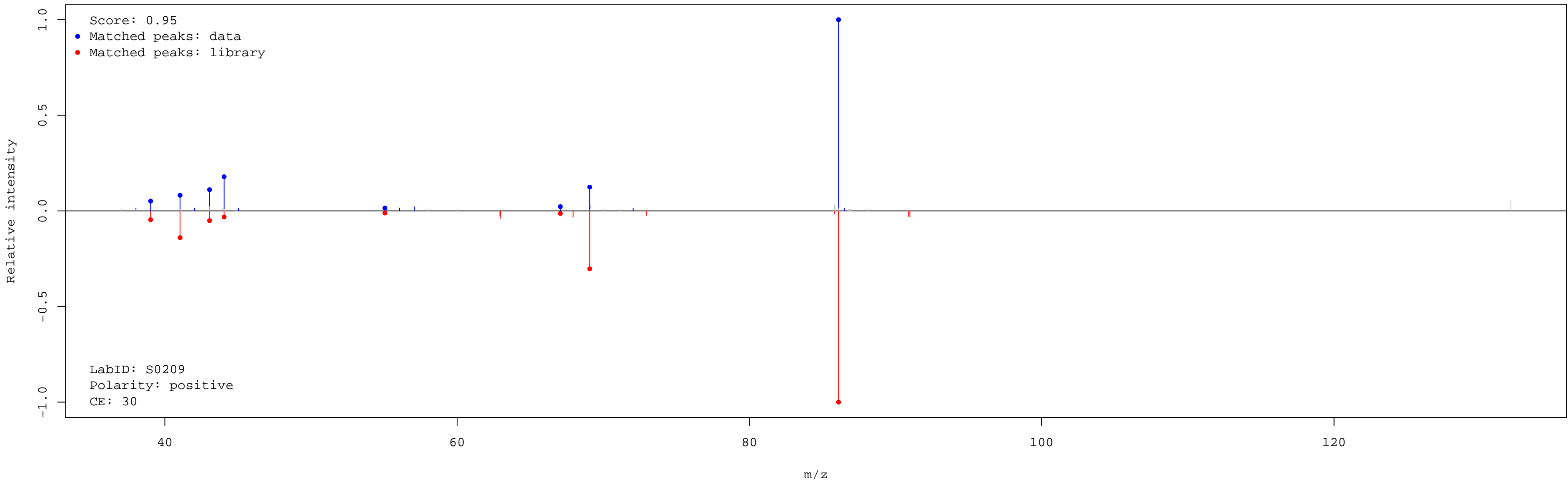

Supplement: Supplementary file 1 — Supplementary Information 1. [file 41598_2021_81109_MOESM1_ESM.zip › 0.95,L-Norleucine,(M+H)+.pdf]

# Corticosterone

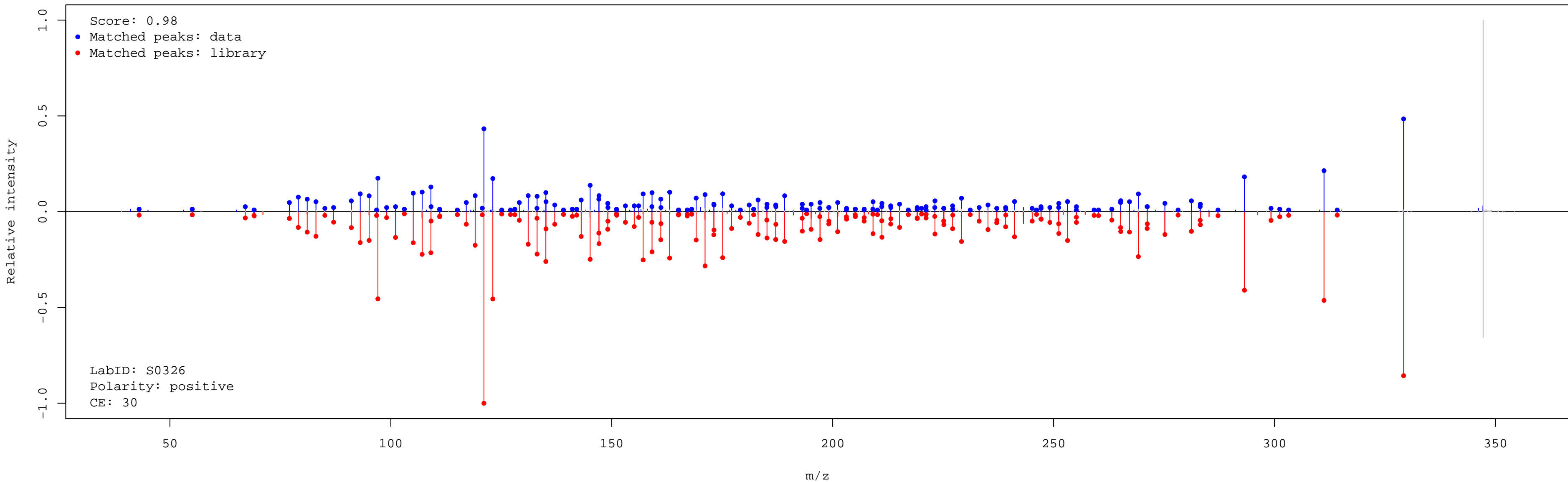

Supplement: Supplementary file 1 — Supplementary Information 1. [file 41598_2021_81109_MOESM1_ESM.zip › 0.98,Corticosterone,(M+H)+.pdf]

# Indoleacrylic acid

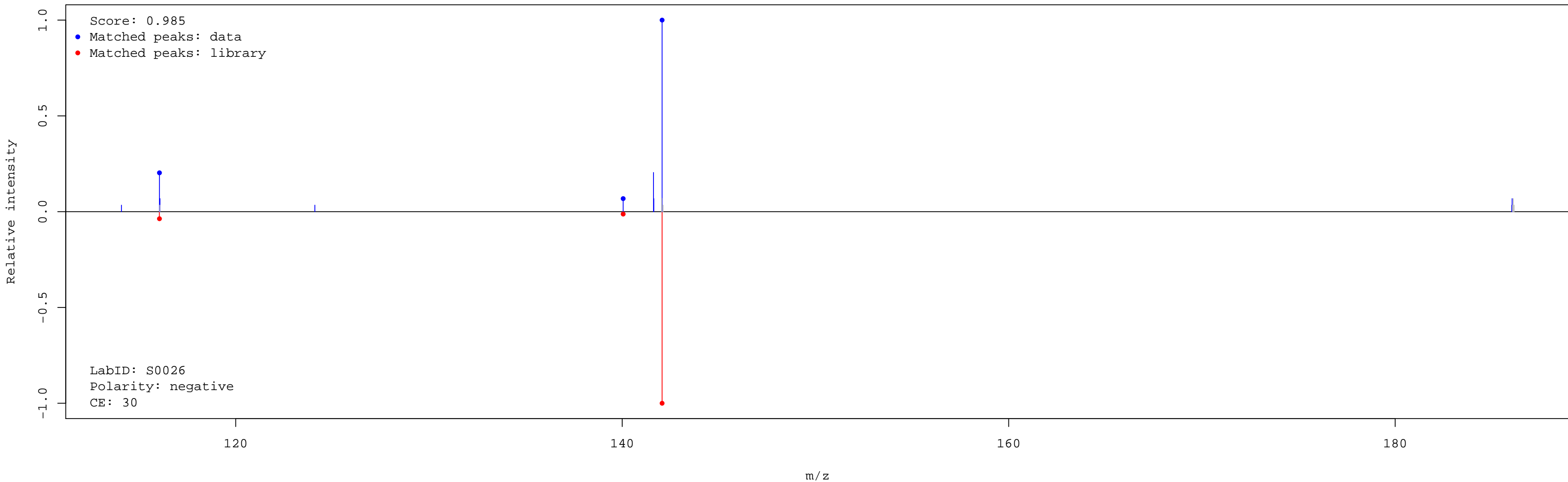

Supplement: Supplementary file 1 — Supplementary Information 1. [file 41598_2021_81109_MOESM1_ESM.zip › 0.985,Indoleacrylic acid,(M-H)-.pdf]

# L-Carnosine

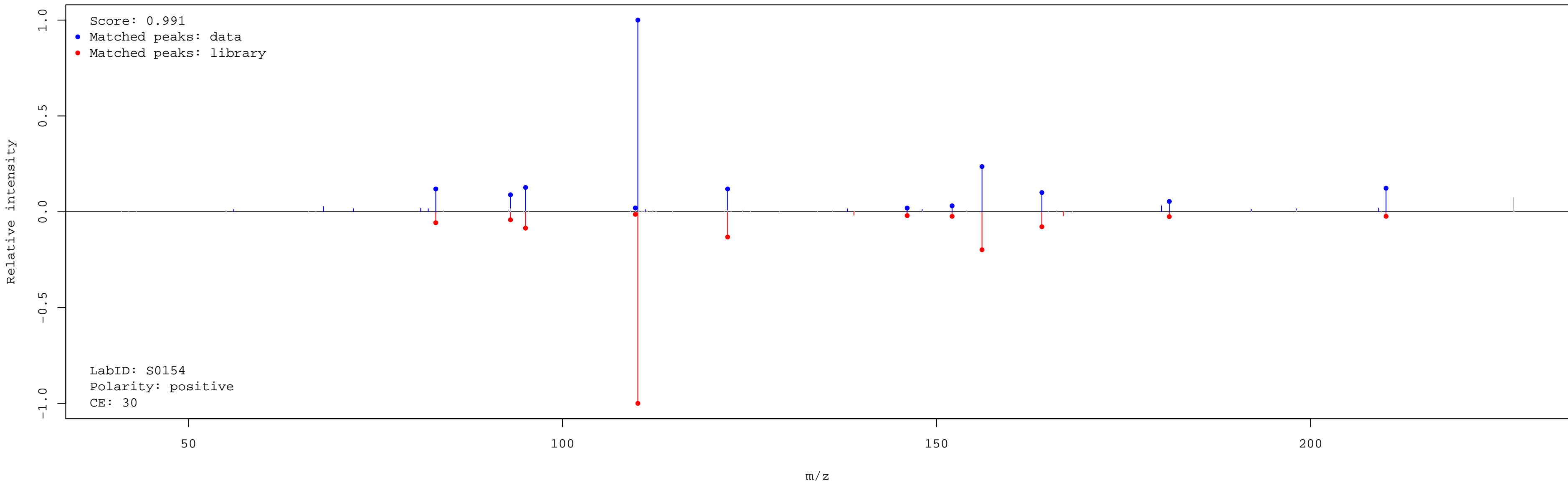

Supplement: Supplementary file 1 — Supplementary Information 1. [file 41598_2021_81109_MOESM1_ESM.zip › 0.991,L-Carnosine,(M+H)+.pdf]

# L-Phenylalanine

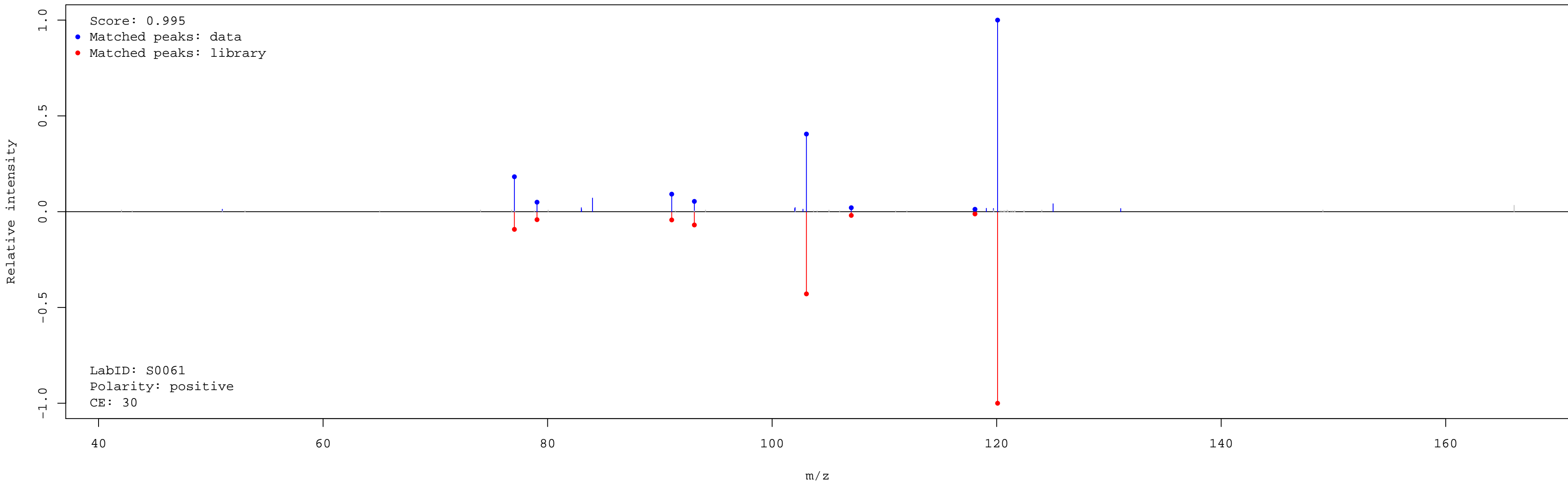

Supplement: Supplementary file 1 — Supplementary Information 1. [file 41598_2021_81109_MOESM1_ESM.zip › 0.995,L-Phenylalanine,(M+H)+.pdf]

# L-Valine

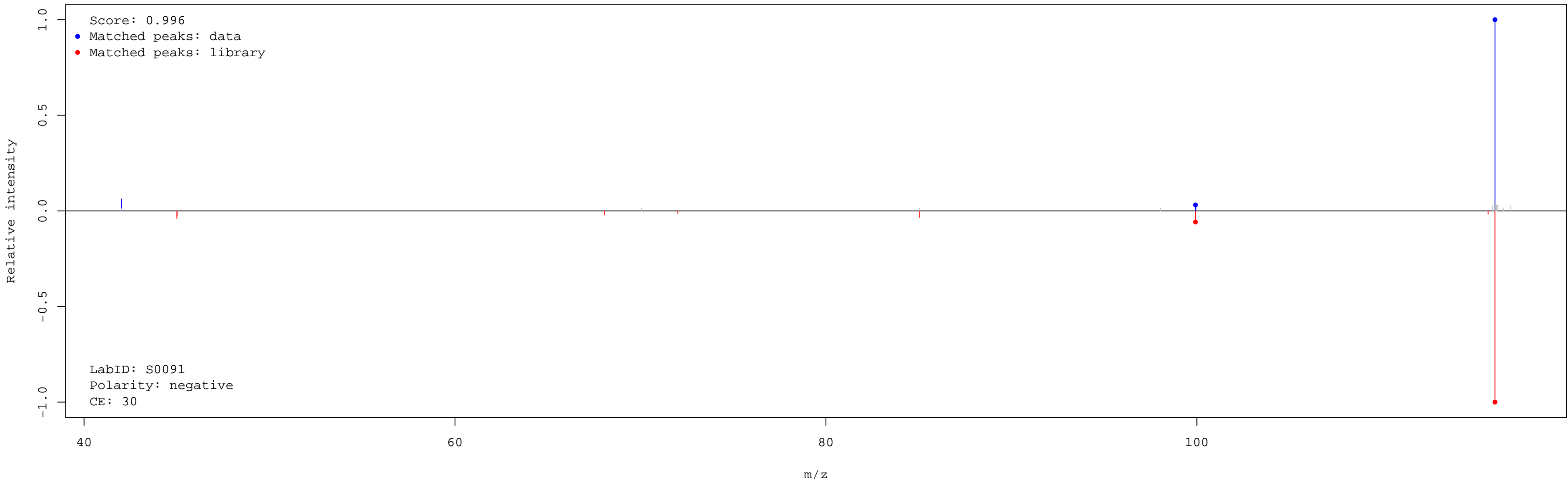

Supplement: Supplementary file 1 — Supplementary Information 1. [file 41598_2021_81109_MOESM1_ESM.zip › 0.996,L-Valine,(M-H)-.pdf]

Nicotinamide

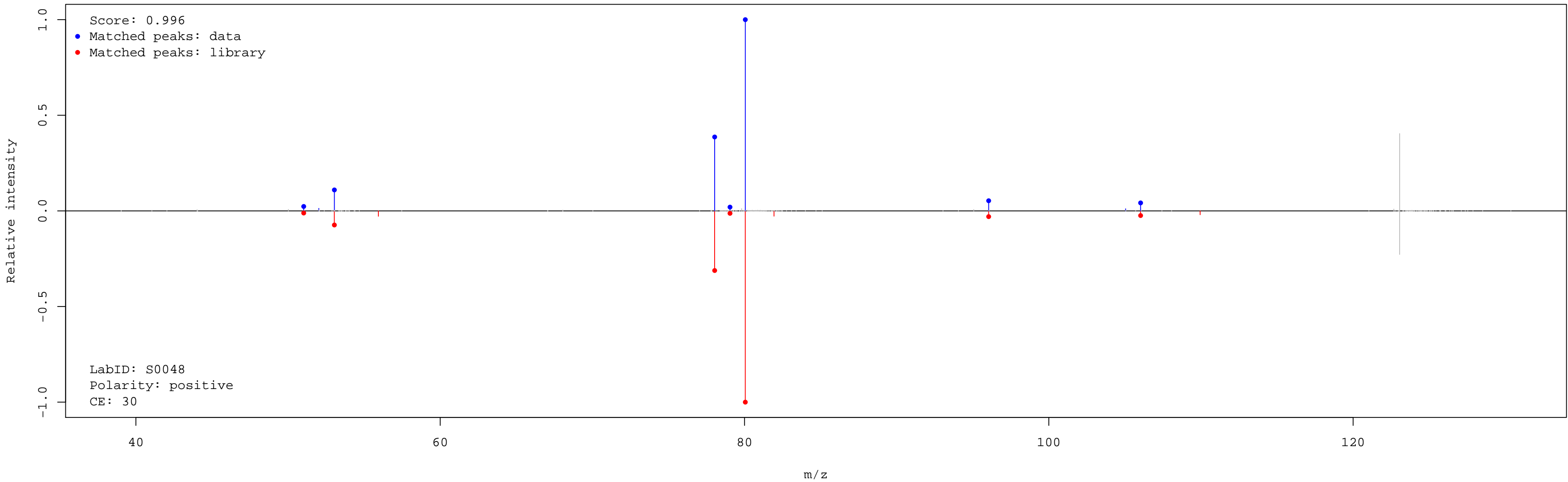

Supplement: Supplementary file 1 — Supplementary Information 1. [file 41598_2021_81109_MOESM1_ESM.zip › 0.996,Nicotinamide,(M+H)+.pdf]

Cholesterol sulfate

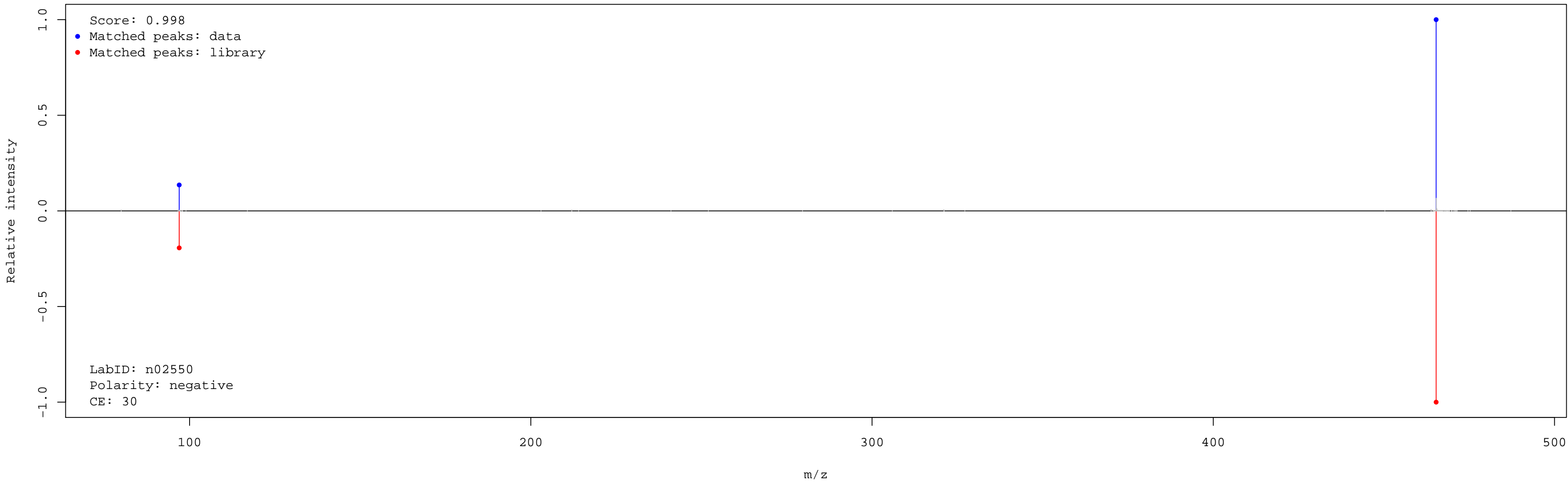

Supplement: Supplementary file 1 — Supplementary Information 1. [file 41598_2021_81109_MOESM1_ESM.zip › 0.998,Cholesterol sulfate,(M-H)-.pdf]

# Creatine

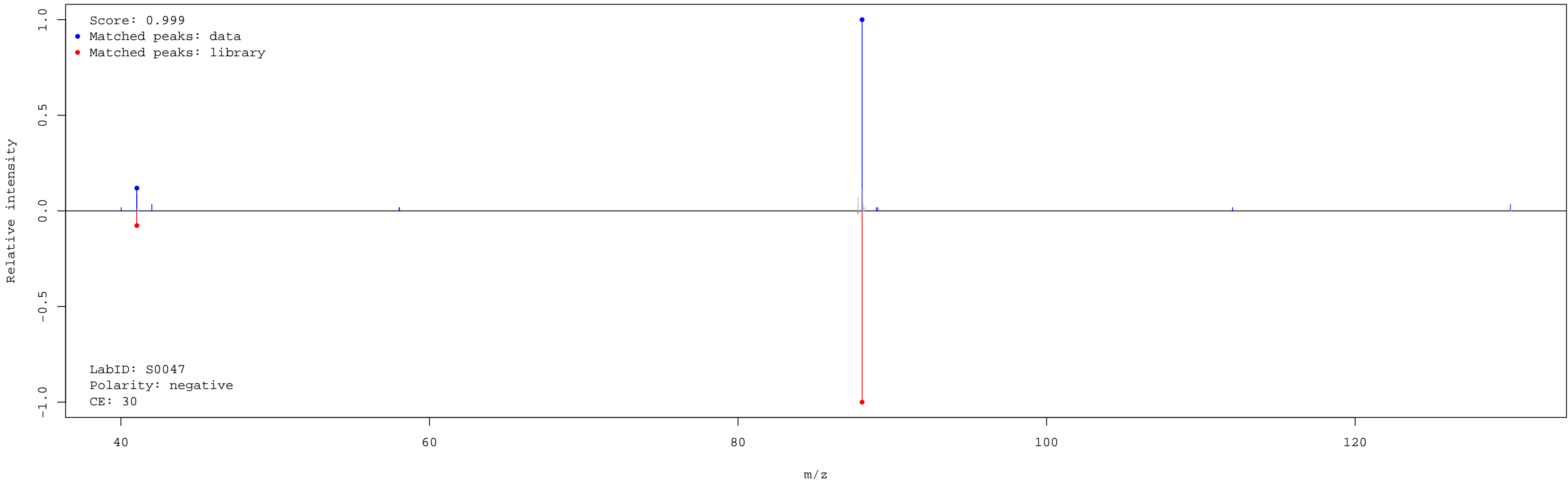

Supplement: Supplementary file 1 — Supplementary Information 1. [file 41598_2021_81109_MOESM1_ESM.zip › 0.999,Creatine,(M-H)-.pdf]

Dodecanoic acid

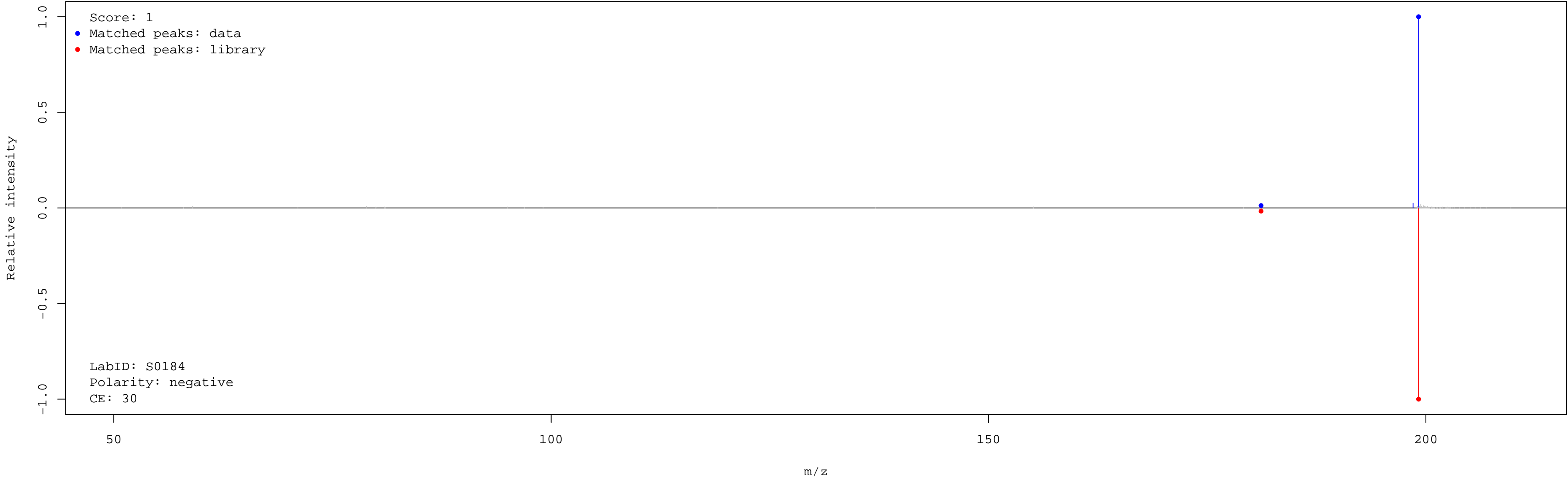

Supplement: Supplementary file 1 — Supplementary Information 1. [file 41598_2021_81109_MOESM1_ESM.zip › 1,Dodecanoic acid,(M-H)-.pdf]

L-Pipecolic acid

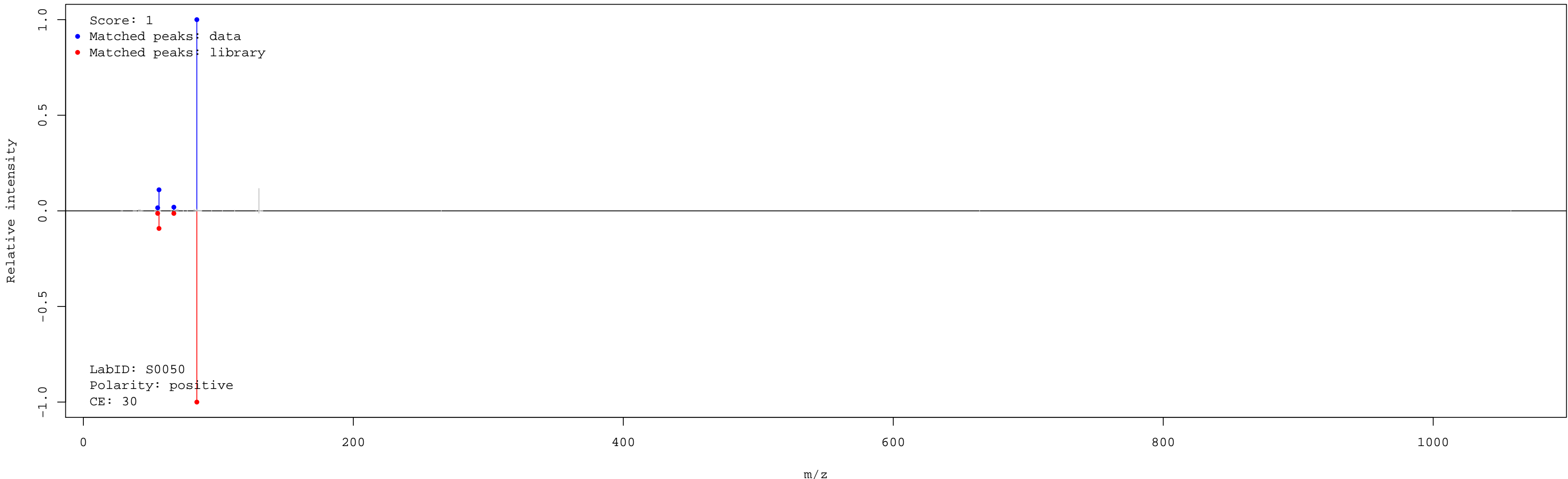

Supplement: Supplementary file 1 — Supplementary Information 1. [file 41598_2021_81109_MOESM1_ESM.zip › 1,L-Pipecolic acid,(M+H)+.pdf]

Taurochenodeoxycholate

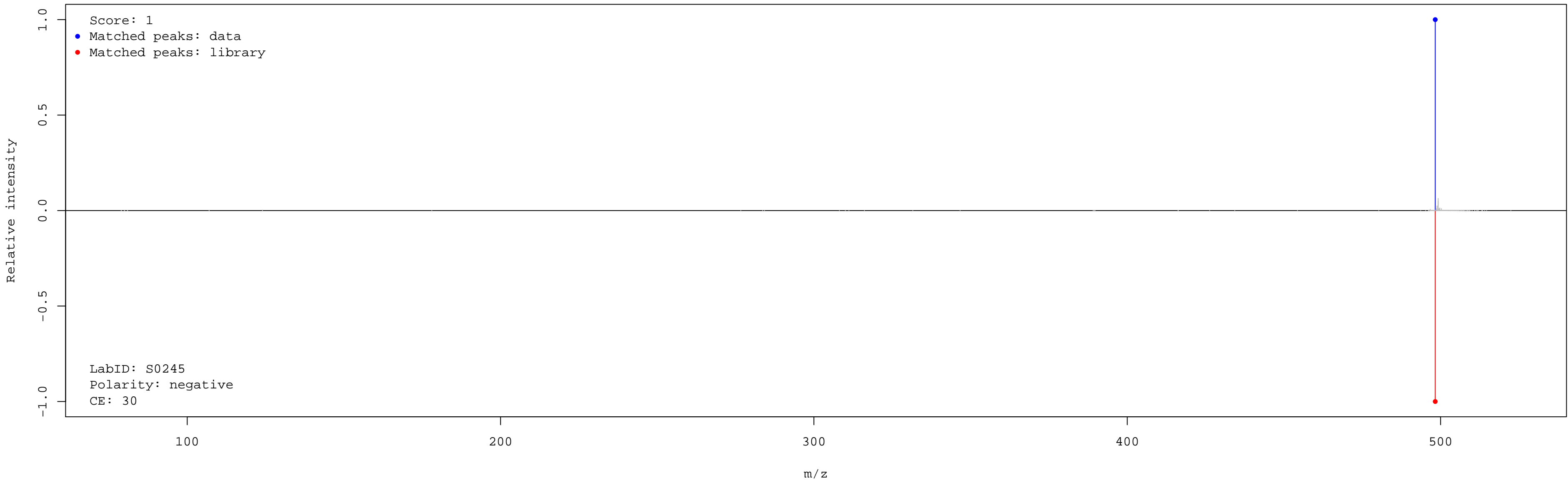

Supplement: Supplementary file 1 — Supplementary Information 1. [file 41598_2021_81109_MOESM1_ESM.zip › 1,Taurochenodeoxycholate,(M-H)-.pdf]

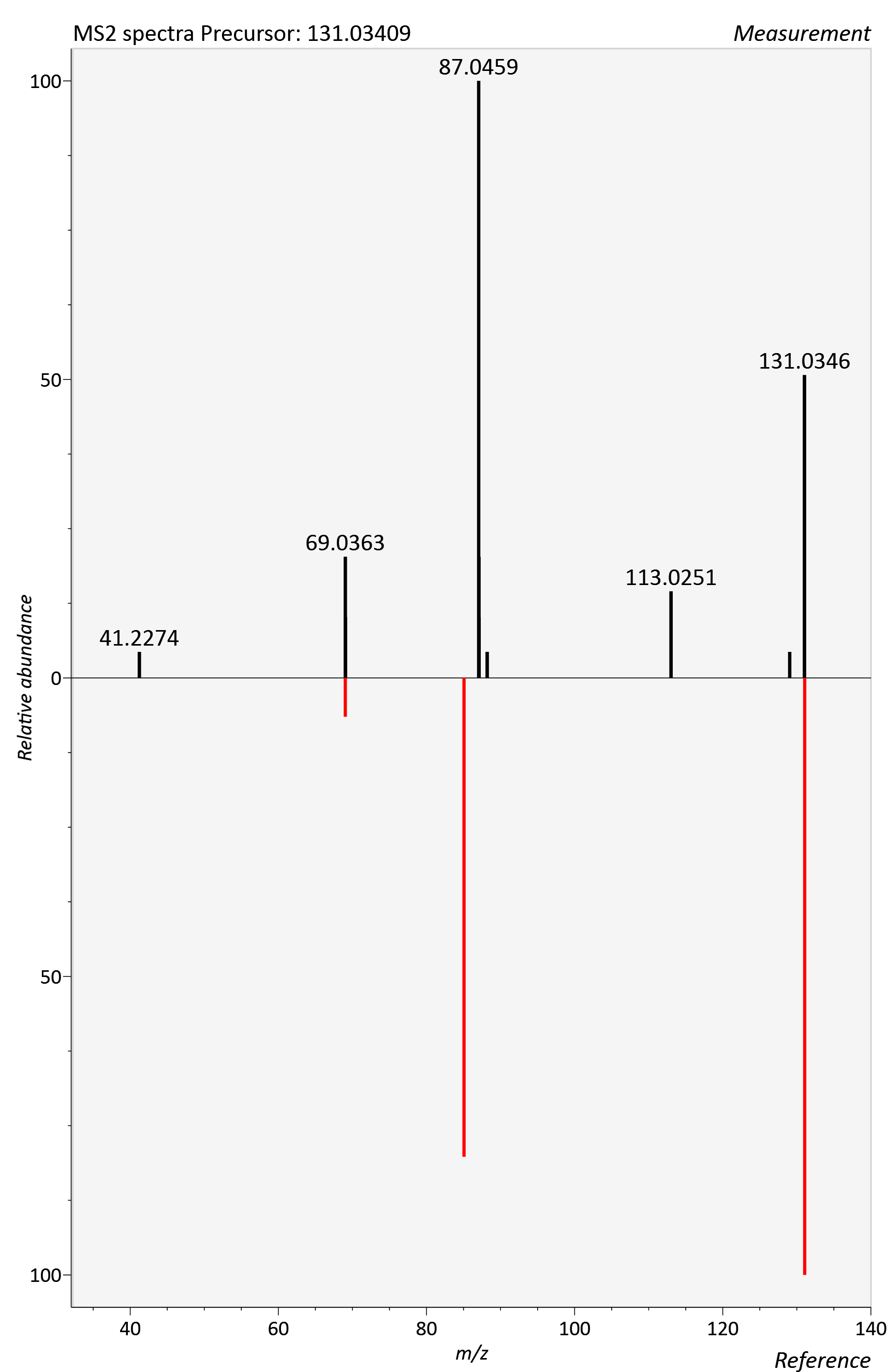

Supplement: Supplementary file 1 — Supplementary Information 1. [file 41598_2021_81109_MOESM1_ESM.zip › 2-Hydroxy-4-methylpentanoate.tiff]

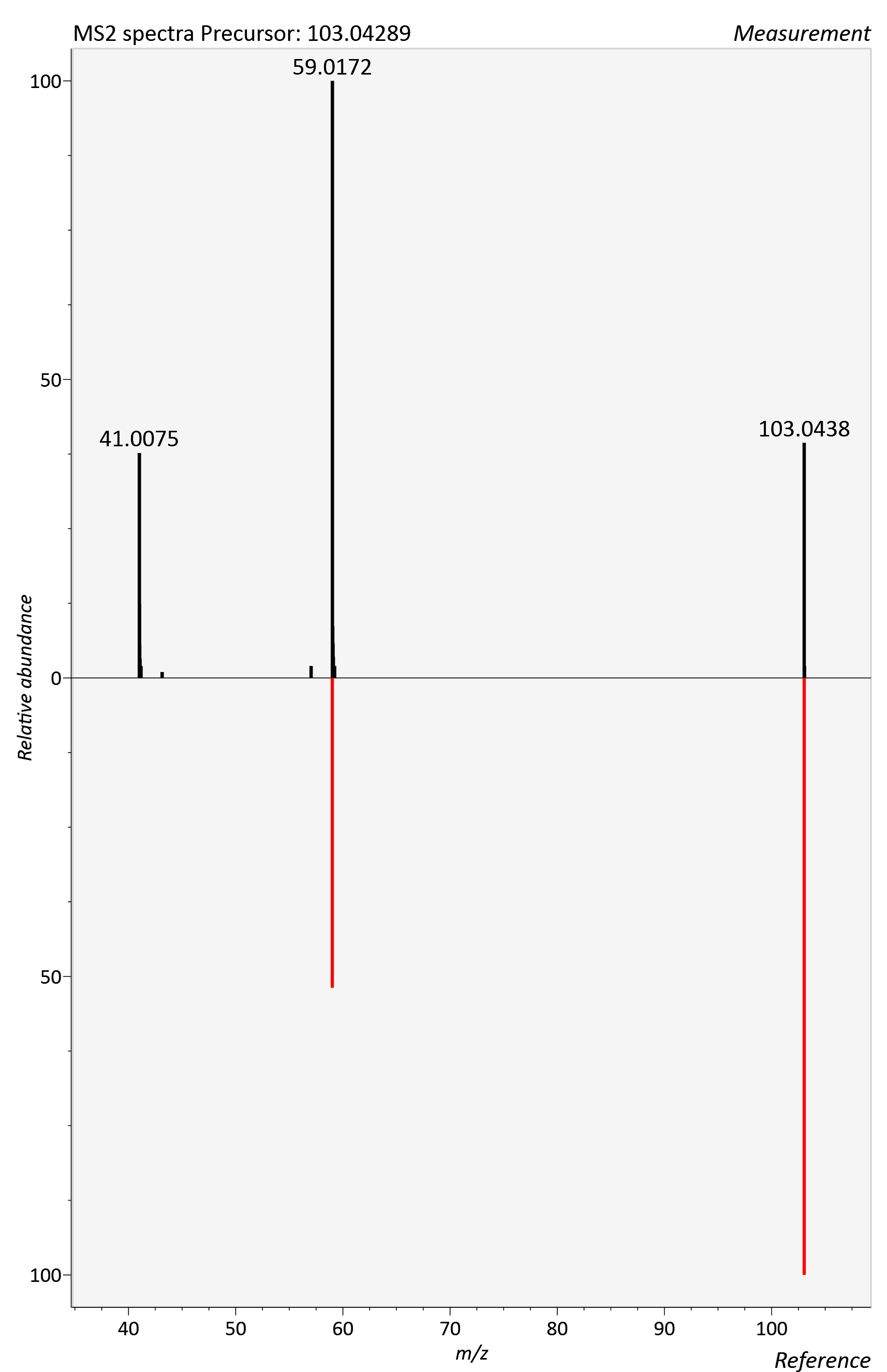

Supplement: Supplementary file 1 — Supplementary Information 1. [file 41598_2021_81109_MOESM1_ESM.zip › DL-beta-Hydroxybutyric acid.tiff]

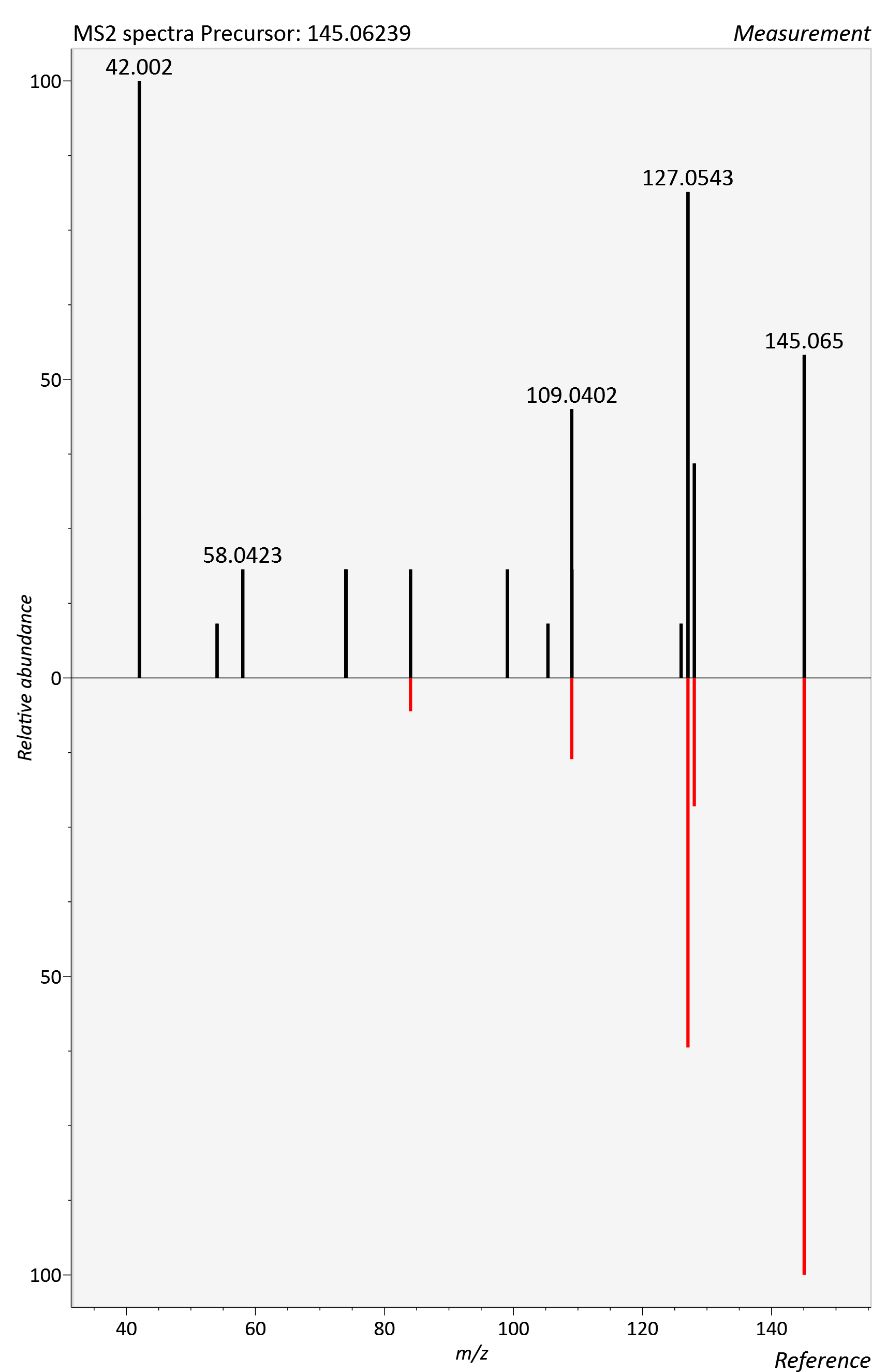

Supplement: Supplementary file 1 — Supplementary Information 1. [file 41598_2021_81109_MOESM1_ESM.zip › Glutamine.tiff]

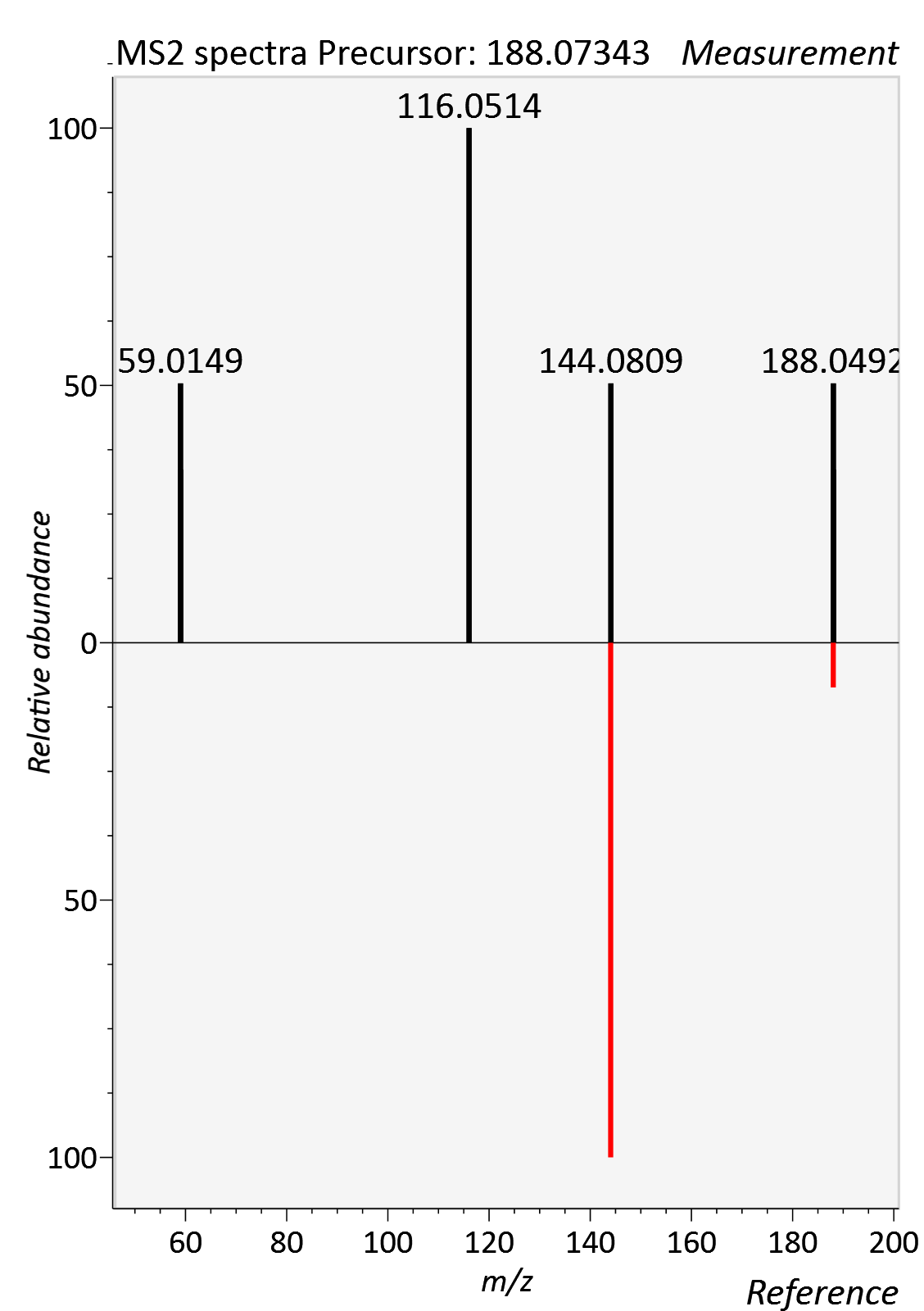

Supplement: Supplementary file 1 — Supplementary Information 1. [file 41598_2021_81109_MOESM1_ESM.zip › Kynurenic acid.tiff]

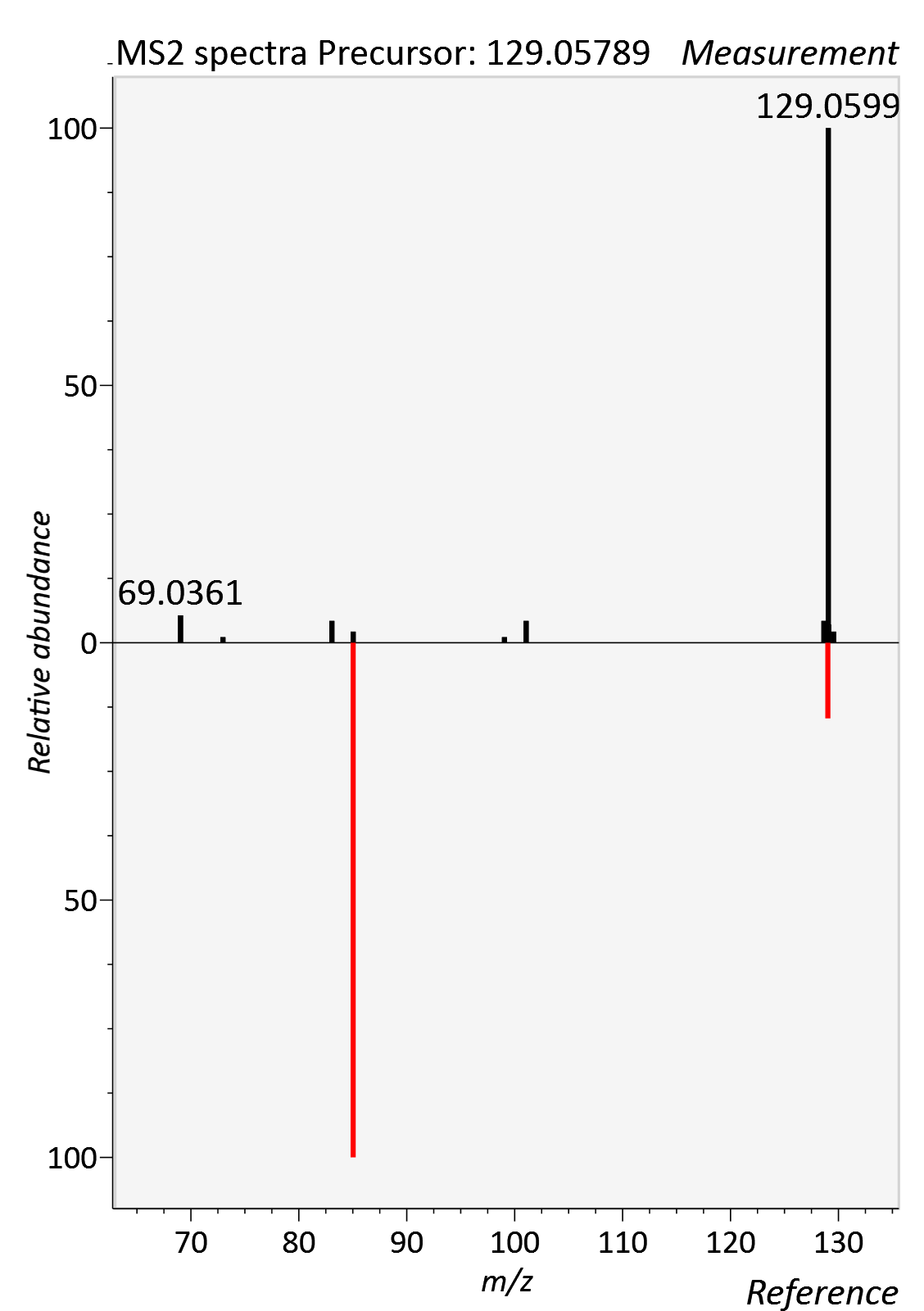

Supplement: Supplementary file 1 — Supplementary Information 1. [file 41598_2021_81109_MOESM1_ESM.zip › Mesaconic acid.tiff]

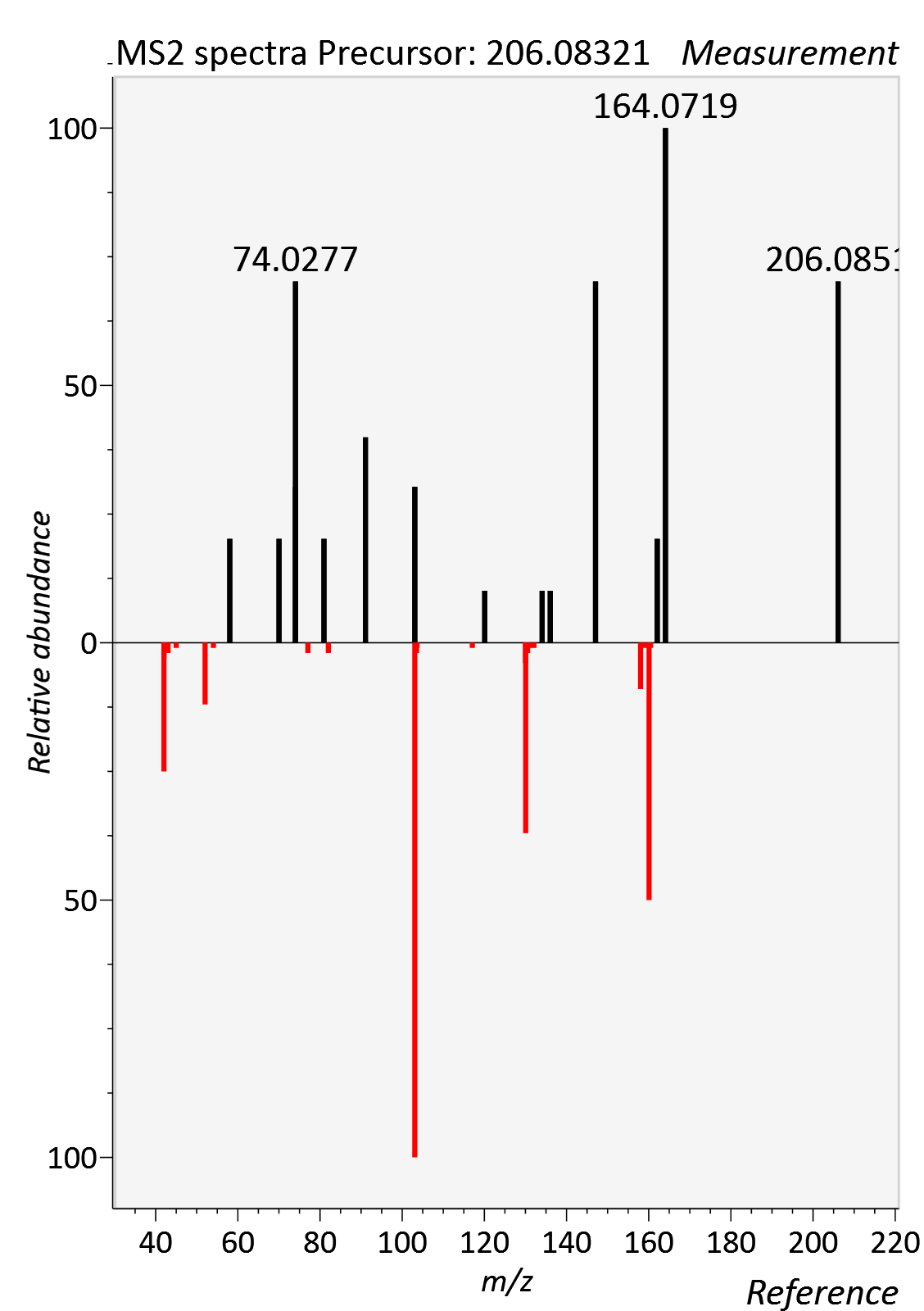

Supplement: Supplementary file 1 — Supplementary Information 1. [file 41598_2021_81109_MOESM1_ESM.zip › N-Cinnamoylglycine.tiff]

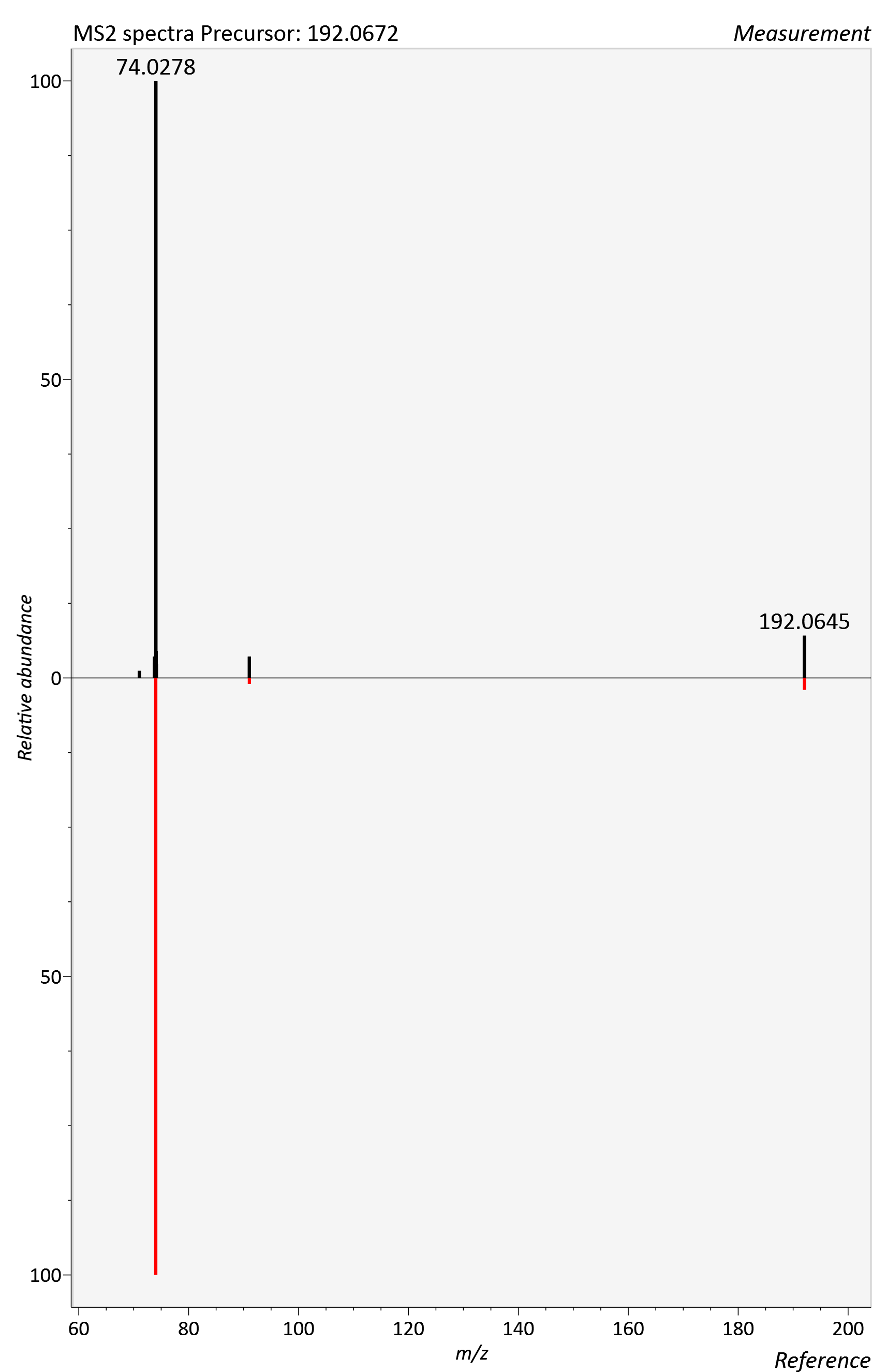

Supplement: Supplementary file 1 — Supplementary Information 1. [file 41598_2021_81109_MOESM1_ESM.zip › Phenylacetylglycine.tiff]

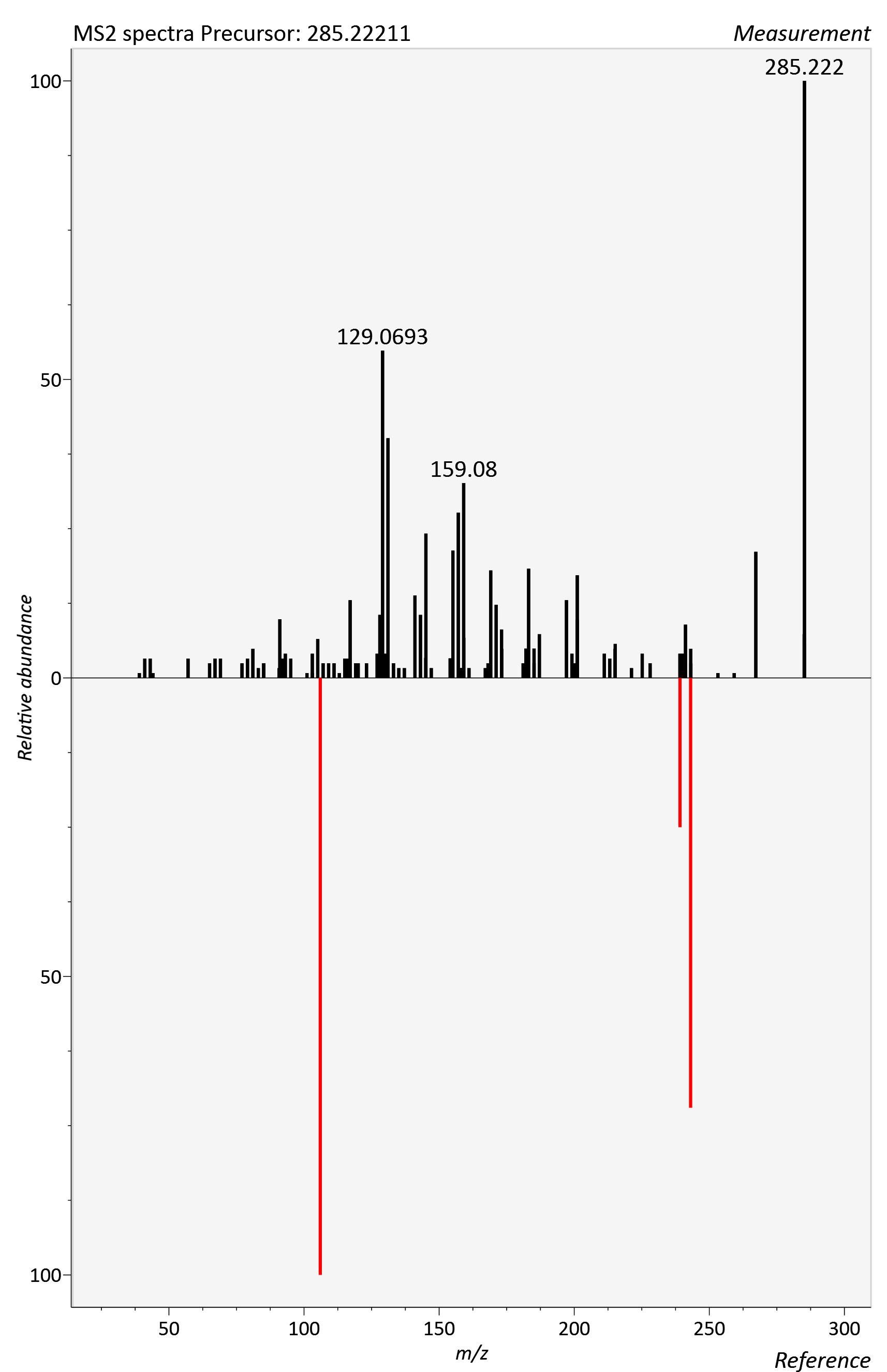

Supplement: Supplementary file 1 — Supplementary Information 1. [file 41598_2021_81109_MOESM1_ESM.zip › acacetin.tiff]

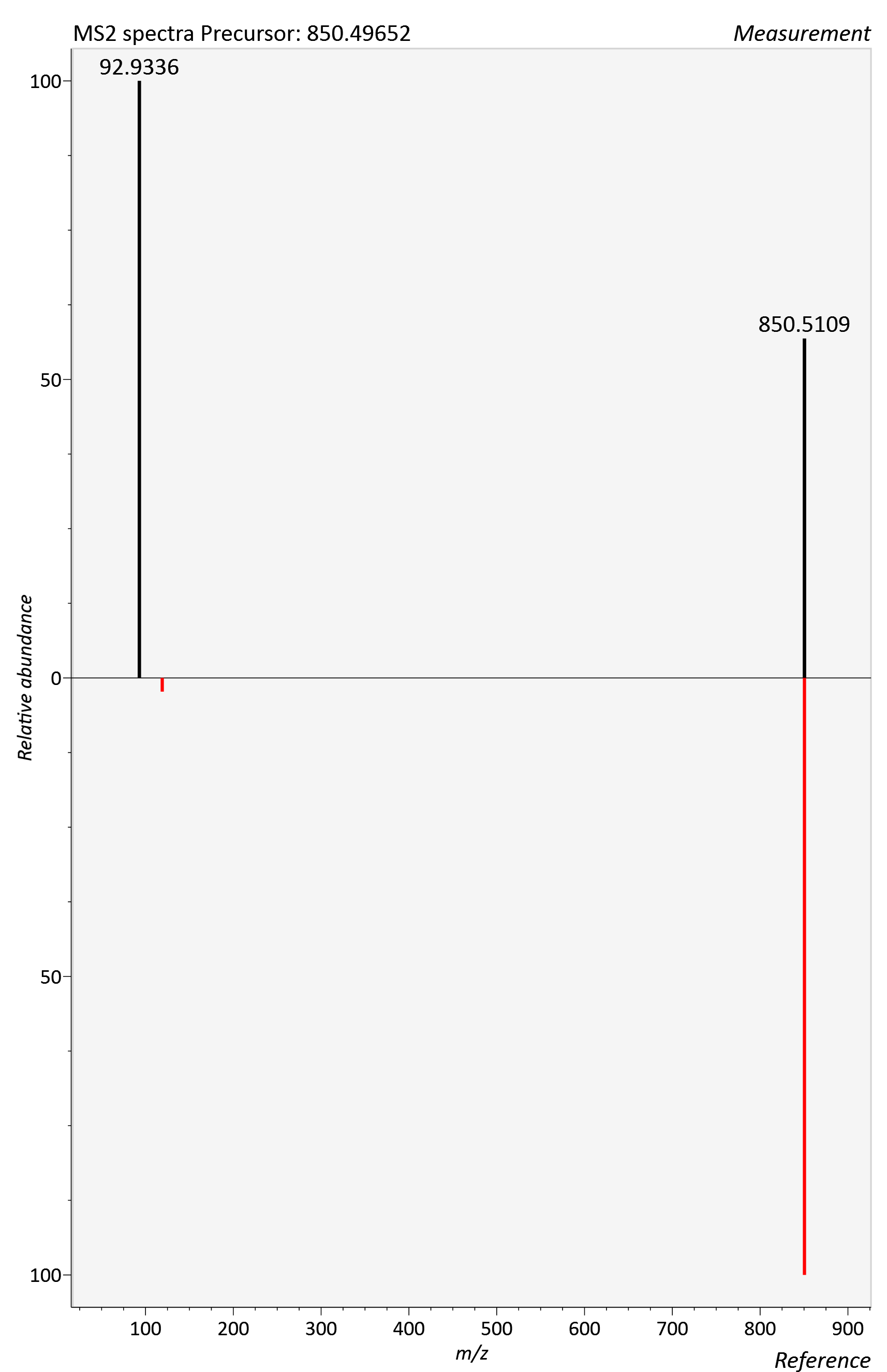

Supplement: Supplementary file 1 — Supplementary Information 1. [file 41598_2021_81109_MOESM1_ESM.zip › alpha-chaconine.tiff]

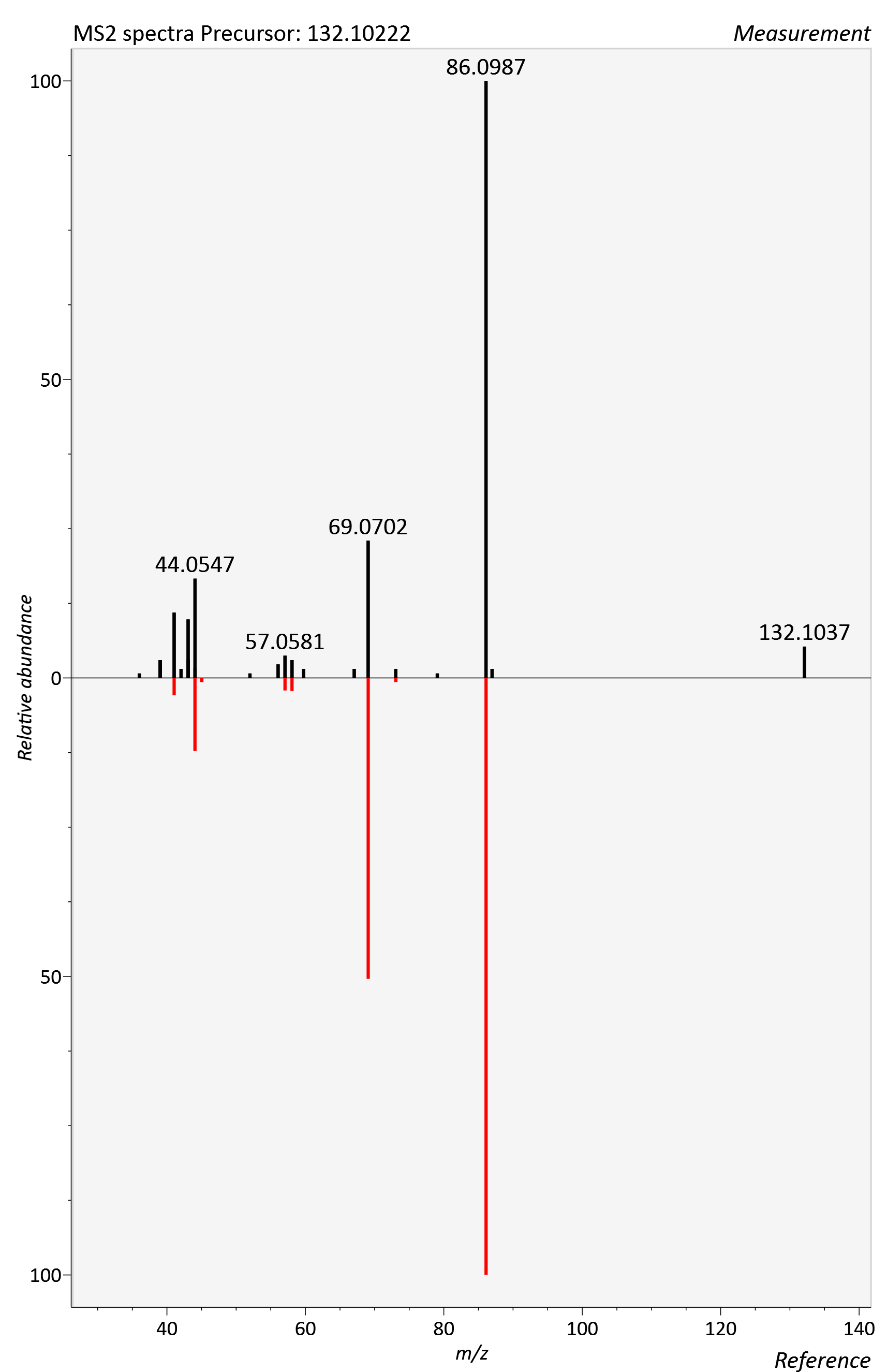

Supplement: Supplementary file 1 — Supplementary Information 1. [file 41598_2021_81109_MOESM1_ESM.zip › isoleucine.tiff]
