# Supplementary material for: Changes on proteomic and metabolomic profile in serum of mice induced by chronic exposure to tramadol
Source: Sci Rep. 2021 Jan 14;11:1454. doi: 10.1038/s41598-021-81109-7 (PMC7809287; doi:10.1038/s41598-021-81109-7)
Supplement: Supplementary file 3 — Supplementary Figure 2. [file 41598_2021_81109_MOESM3_ESM.docx]

Changes on proteomic and metabolomic profile in serum of mice induced by chronic exposure to tramadol

Shukun Jiang#^1^,Guojie Liu #^2^, Huiya Yuan^1^,Enxu Yu^1^,Wei Xia^1^, Xiaoyu Zhang^3^, Junting Liu^1^，Lina Gao^1*^

( 1School of Forensic Medicine, China Medical University, Shenyang, 110014, China;

1. School of Fundamental Sciences,China Medical University, Shenyang, 110014, China;
2. Jacobs University, Bremen, 28759, Germany)


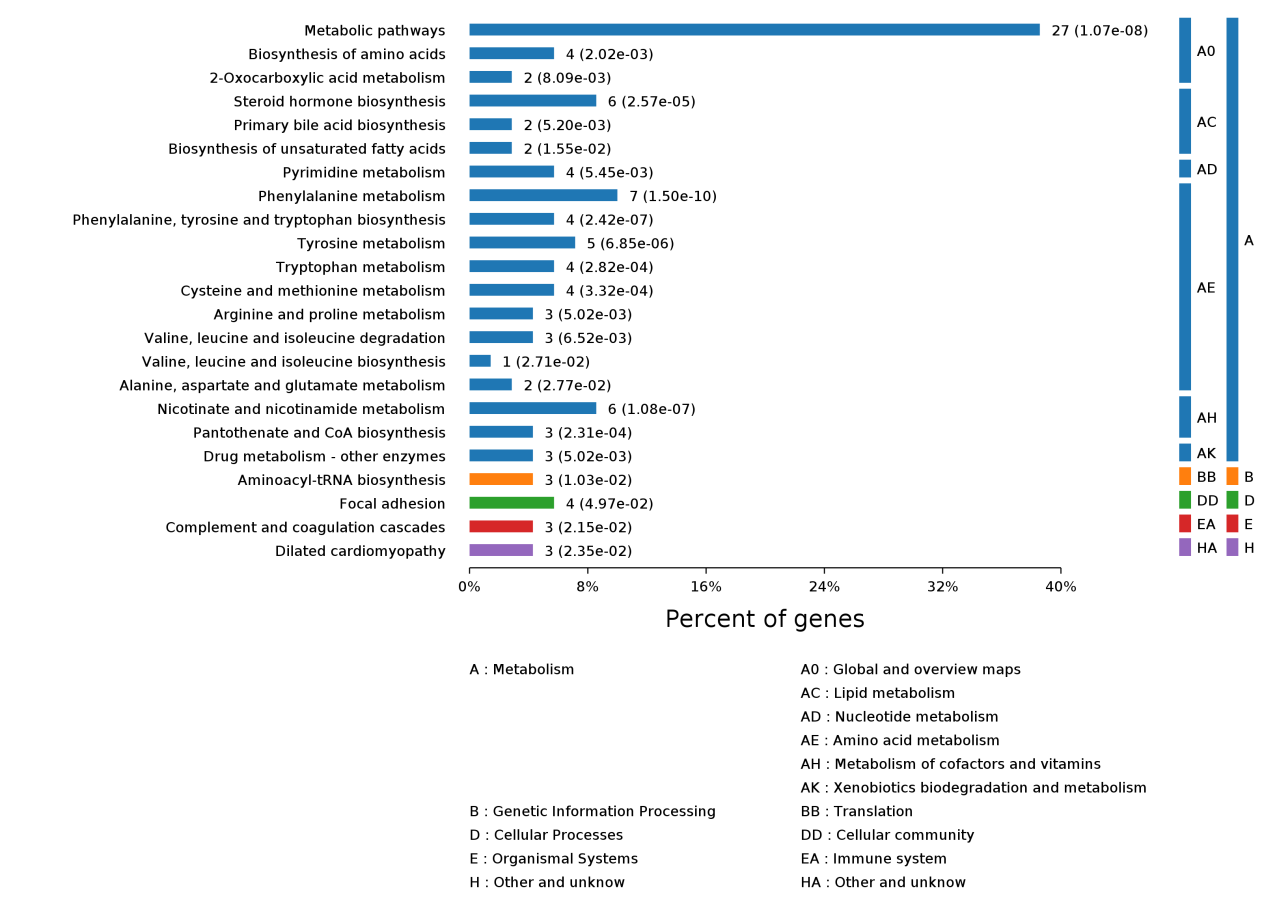


Supplementary Figure2. Integrated analysis of proteomics and metabolomics combination of KEGG metabolic pathway enrichment -H vs N
